# TRIM56 Promotes White Adipose Tissue Browning to Attenuate Obesity by Degrading TLE3

**DOI:** 10.1002/advs.202414073

**Published:** 2025-02-10

**Authors:** Haojie Qin, Yi Zhong, Jinhui Huang, Yanli Miao, Meng Du, Kai Huang

**Affiliations:** ^1^ Clinic Center of Human Gene Research Union Hospital Tongji Medical College Huazhong University of Science and Technology Wuhan 430022 China; ^2^ Department of Cardiology Union Hospital Tongji Medical College Huazhong University of Science and Technology Wuhan 430022 China; ^3^ Department of Rheumatology and Immunology Union Hospital Tongji Medical College Huazhong University of Science and Technology Wuhan 430022 China; ^4^ Department of Cardiology The First Affiliated Hospital of Zhengzhou University Zhengzhou 450052 China; ^5^ Hubei Key Laboratory of Metabolic Abnormalities and Vascular Aging Huazhong University of Science and Technology Wuhan 430022 China; ^6^ Hubei Clinical Research Center of Metabolic and Cardiovascular Disease Huazhong University of Science and Technology Wuhan 430022 China

**Keywords:** adaptive thermogenesis, Lipolysis, obesity, ubiquitin modifications, White adipose tissue browning

## Abstract

In mammals, the activation of thermogenic adipocytes, such as beige and brown adipocytes, can significantly increase overall energy expenditure, offering a promising strategy to combat metabolic diseases. Despite its considerable potential, the regulatory mechanisms governing this activation remain largely elusive. This study bridges this gap by elucidating that tripartite motif 56 (TRIM56), an E3 ubiquitin ligase, is upregulated in response to cold stimuli, thereby promoting the recruitment of beige adipocytes. Notably, the overexpression of TRIM56 in adipocytes is shown to help mice maintain a core temperature under cold conditions, as well as confer protection against diet‐induced obesity. Mechanistically, TRIM56 facilitates the degradation of the transducin‐like enhancer protein 3 (TLE3) protein by promoting its K48‐linked ubiquitination, which subsequently triggers the activation of thermogenic genes in subcutaneousl white adipose tissue and improved the metabolic profiles. These findings unveil a novel function for TRIM56 in adipocyte browning, suggesting its potential as a therapeutic target for the treatment of metabolic disorders.

## Graphical abstract

This study pinpoints TRIM56 as a viable candidate for promoting white adipose tissue browning and as a promising therapeutic target for addressing obesity and metabolic disorders. Functioning as an E3 ubiquitin ligase, TRIM56 facilitates the degradation of the TLE3 protein, thereby accelerating the thermogenic process and enhancing energy expenditure. This Figure was created with BioRender.com/g97l202.

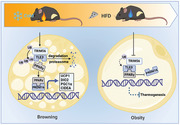

## 1 Introduction

Obesity stands as a crucial risk factor for various diseases, such as metabolic‐associated fatty liver disease (MAFLD), type 2 diabetes and cardiovascular disease, emerging as a severe health challenge on a global scale.^[^ ^1^, ^2^, ^3^, ^4^, ^5^, ^6^ ^]^ Additionally, it is estimated that over 4 million annual deaths are linked to excess body weight.^[^ ^7^, ^8^ ^]^ The etiology of obesity is rooted in the disparity between caloric intake and metabolic expenditure, necessitating interventions that focus on reducing caloric intake and enhancing energy expenditure.^[^ ^9^, ^10^, ^11^, ^12^ ^]^

Adipose tissue stands as a key organ in metabolic regulating, featuring a complex micro‐environment and multifunctional roles.^[^ ^10^, ^11^ ^]^ In humans, two primary types of adipose tissue have been recognized: white adipose tissue (WAT) and brown adipose tissue (BAT).^[^ ^13^, ^14^ ^]^ WAT, characterized by lipid‐storing adipocytes, is essential for energy conservation. Conversely, BAT, marked by elevated uncoupling Protein 1 (UCP1) content in adipocytes, is specialized for thermogenic energy dissipation. Exposure to cold conditions or β3‐adrenergic agonists triggers the expression of thermogenic genes in white adipocytes,^[^ ^3^ ^]^ thereby transforming them into beige adipocytes with heat generating properties similar to brown adipocytes. This metabolic reprogramming of white adipocytes, termed “browning”, is a promising approach to enhance thermogenic capacity and ameliorate obesity.^[^ ^5^, ^13^ ^]^

Tripartite motif (TRIM) proteins, a large family of E3 ubiquitin ligases, are characterized by a tripartite domain composed of a RING finger, one or two zinc‐binding motifs and a coiled‐coil region.^[^ ^15^, ^16^, ^17^ ^]^ Previous reports have shown that TRIM proteins are involved in a great range of cellular processes, such as proliferation, differentiation, tumorigenesis, autophagy and immunity.^[^ ^15^, ^16^, ^18^ ^]^ However, the investigation of TRIM proteins in the context of metabolic dysfunction remains confined to merely a subset of its members, leaving much territory yet to be explored.

TRIM56, also referred to as RNF109, is involved in diverse physiological and pathological processes by ubiquitinating an array of substrates.^[^ ^15^, ^19^, ^20^, ^21^ ^]^ Previous research indicates that the activation of TRIM56 enhances antiviral responses, alleviates MAFLD,^[^ ^22^ ^]^ and inhibits the growth of several cancers, including multiple myeloma, hepatocellular carcinoma, lung adenocarcinoma, and leukemia.^[^ ^23^ ^]^ Conversely, TRIM56 can also facilitate the progression of glioma, breast cancer, and Kaposi's sarcoma.^[^ ^18^, ^24^, ^25^ ^]^ The multifaceted roles underscore the complexity of TRIM56's functions and highlight its potential for clinical applications, despite not yet being utilized as a clinical target.

Our research has expanded the understanding of TRIM56's role in metabolic disorders. Here, we found that TRIM56 is selectively regulated in WAT by thermal or metabolic pressure, and promotes adipocytes thermogenesis by degrading TLE3. Notably, enforcing TRIM56 expression in WAT could help mice maintain body temperature in cold environments and ameliorate diet‐induced obesity, suggesting its potential as a novel target for metabolic disease management.

## 2 Results

### 2.1 TRIM56 is Upregulated under Thermal Stress but Downregulated in Diet‐Induced Obesity

To investigate the role of TRIM proteins in adaptive thermogenesis of adipose tissue, we first analyzed the expression changes of TRIM proteins in response to cold exposure or after CL316243 (a β3 adrenoceptor agonist) treatment using publicly available single‐cell datasets (GSE133486).^[^ ^26^ ^]^ We found that prolonged cold stimulation induces a significant upregulation of TRIM44 and TRIM56, which is more pronounced than other members, in adipocytes of inguinal white adipose tissue (iWAT). However, in the CL316343‐treated group, the expression of TRIM44 remained unchanged, while the expression of TRIM56 increased, which is consistent with that under cold conditions (Figure S1A, Supporting Information). Therefore, we hypothesize that TRIM56 may play an important role in thermogenesis and temperature maintenance in mice.

**Figure 1:**
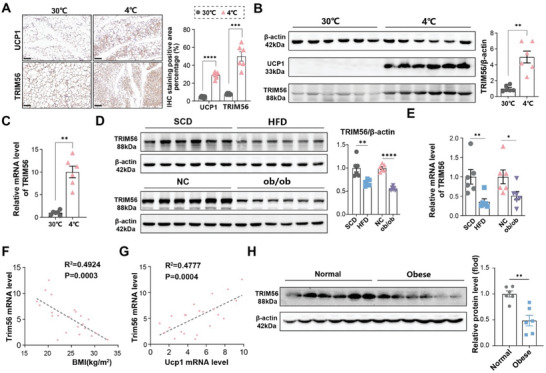
TRIM56 expression was upregulated by cold stress but downregulated in obesity. A–C) After housed in 30 °C for 2 weeks, C57BL/6J mice were transferred to 4 °C or maintained at 30 °C for 3 days (*n* = 6 per group). A), Representative immunohistochemical images of UCP1 and TRIM56 (left), with quantification (right). Scale bar,100 µm. B), Representative Immunoblot analysis of β‐actin, UCP1 and TRIM56 proteins in iWAT (left), with quantification (right). C) RT‐qPCR analysis of TRIM56 mRNA level in iWAT. D) Immunoblot analysis of β‐actin and TRIM56 proteins (left) in iWAT from C57BL/6J mice subjected to a 12‐week HFD and ob/ob mice, wild type mice subjected to a SCD were used as controls, with their quantification (right, *n* = 6 per group). E) RT‐qPCR analysis of TRIM56 levels in iWAT from mice mentioned above (*n* = 6 per group). F,G) Linear regression analyses were conducted to examine the relationship between TRIM56 mRNA levels and BMI (F), as well as with UCP1 mRNA levels (G), in human abdominal adipose tissues. Each data point represents an individual participant (*n* = 22). H) Immunoblot analysis of β‐actin, and TRIM56 proteins (left), with quantification (right) in iWAT from human abdominal fat (*n* = 6 per group). Data are presented as mean ± SEM. *, *P* < 0.05, **, *P* < 0.01, ***, *P* < 0.001, and ****, *P* < 0.0001. ns, non‐significant. HFD, high fat diet. SCD, standard control diet. NC, normal control.

Our initial investigations revealed widespread expression of TRIM56 across various tissues, with particularly pronounced levels detected in both BAT and WAT (Figure S1B, Supporting Information). To further explore the role of TRIM56 in the browning process of WAT, we assessed TRIM56 expression in iWAT subjected to two thermogenic stimuli: exposure to cold temperatures (4 °C for three days) and a seven‐day treatment with CL316243. Both conditions led to significant increases in TRIM56 protein and mRNA levels (**Figure** **1**B,C; Figure S1F,G, Supporting Information). Similar results were observed in mature adipocytes differentiated from stromal vascular fraction cells (SVF) under CL316243 stimulation (Figure S1C,D, Supporting Information). These findings were further verified by immunohistochemical analysis (Figure 1A; Figure S1E, Supporting Information), conclusively demonstrating an upregulation of TRIM56 in response to thermogenic stimuli.

Conversely, in models of diet‐induced obesity, we observed a decrease in both protein and mRNA levels of TRIM56 in iWAT (Figure 1D,E). However, in all of the above mouse models, we did not observe these changes in BAT and epididymal white adipose tissue (eWAT) (Figure S1H–K, Supporting Information). Besides, this trend was also observed in human samples (Figure 1H). Our data revealed a positive correlation between TRIM56 expression and UCP1, a key marker of thermogenesis, and a negative correlation with body mass index (BMI) (Figure 1F,G). Based on these data, we hypothesized that TRIM56 might serve as a potential regulator in adipocytes thermogenesis.

### 2.2 TRIM56 Potentiates the Activation of Beige Adipocytes in Response to β3‐adrenergic Agonists

**Figure 2:**
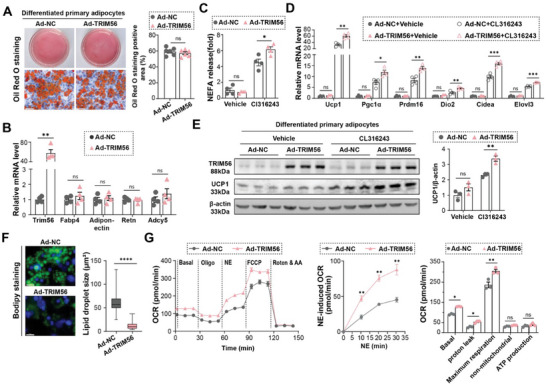
TRIM56 overexpression enhanced CL316243‐induced activation of beige adipocytes in vitro. SVF cells were isolated from the iWAT of C57BL/6J mice and differentiated into mature adipocytes over 8 days. Mature adipocytes were then treated with adenovirus vectors overexpressing TRIM56 or controls. After 24 h of treatment, the cells were stimulated with 1 µm CL316243 for an additional 24 h. A) Represented Oil Red O (ORO) red staining images of mature primary adipocytes, which treated with TRIM56‐overexpressed adenovirus vectors or controls for 24 h, with quantification of ORO staining positive area (right, *n* = 6 per group). Scale bar, 50 um. B) RT‐qPCR analysis of TRIM56 mRNA levels and specified adipocyte markers (*n* = 4). C) NEFA levels in the culture medium (*n* = 4). D) RT‐qPCR analysis of key thermogenic genes (*n* = 4). E) Represented immunoblotting pictures of UCP1, TRIM56 and β‐actin proteins (left) with corresponding quantitative analysis (right, *n* = 3). F) Bodipy staining images of adenovirus‐treated adipocytes after 24 h of CL316243 stimulation with quantification of lipid droplet size (right, Ad‐NC: *n* = 79; Ad‐TRIM56: *n* = 162). Scale bar, 20 um. G) Measurement of oxygen consumption rates (OCR) in mature primary adipocytes subjected to different stimulations (*n* = 3). Data are expressed as mean ± SEM. *, *P* < 0.05, **, *P* < 0.01, ***, *P* < 0.001, and ****, *P* < 0.0001. ns, non‐significant.

We initially examined whether overexpression of TRIM56 could autonomously activate the thermogenic gene program. Using adenovirus vectors, we manipulated the levels of TRIM56 in mature beige adipocytes derived from SVF cells. Modulating TRIM56 expression did not affect lipid storage or adipogenic differentiation (**Figure** **2**A,B; Figure S2A,B, Supporting Information).

CL316243 treatment significantly increased the expression of thermogenic markers (*Ucp1, Prdm16, Cidea, Pgc1α, Dio2*, and *Elvol3*) and raised non‐esterified fatty acid (NEFA) levels in the culture medium. Notably, these effects were further augmented by overexpression of TRIM56, whereas they were attenuated when TRIM56 was knocked down (Figure 2C–E; Figure S2C–E, Supporting Information).

Bodipy staining revealed that, under CL316243 stimulation, overexpression of TRIM56 led to the formation of small, multilocular lipid droplets, a characteristic morphology of beige adipocytes (Figure 2F). Conversely, reducing TRIM56 levels resulted in the opposite phenotype (Figure S2F, Supporting Information).

Additionally, seahorse bioenergetics analysis provided insights into TRIM56's role in mitochondrial respiration. Specifically, overexpressing TRIM56 increased the oxygen consumption rate (OCR), whereas knocking down TRIM56 decreased OCR (Figure 2G; Figure S2G, Supporting Information).

Overall, these findings indicate that TRIM56 in adipocytes functions autonomously to potentiate the activation of the thermogenic gene program induced by β3‐adrenergic receptor agonists.

### 2.3 TRIM56 Overexpression Promotes iWAT Browning and Enhances Lipolysis In Vivo

**Figure 3:**
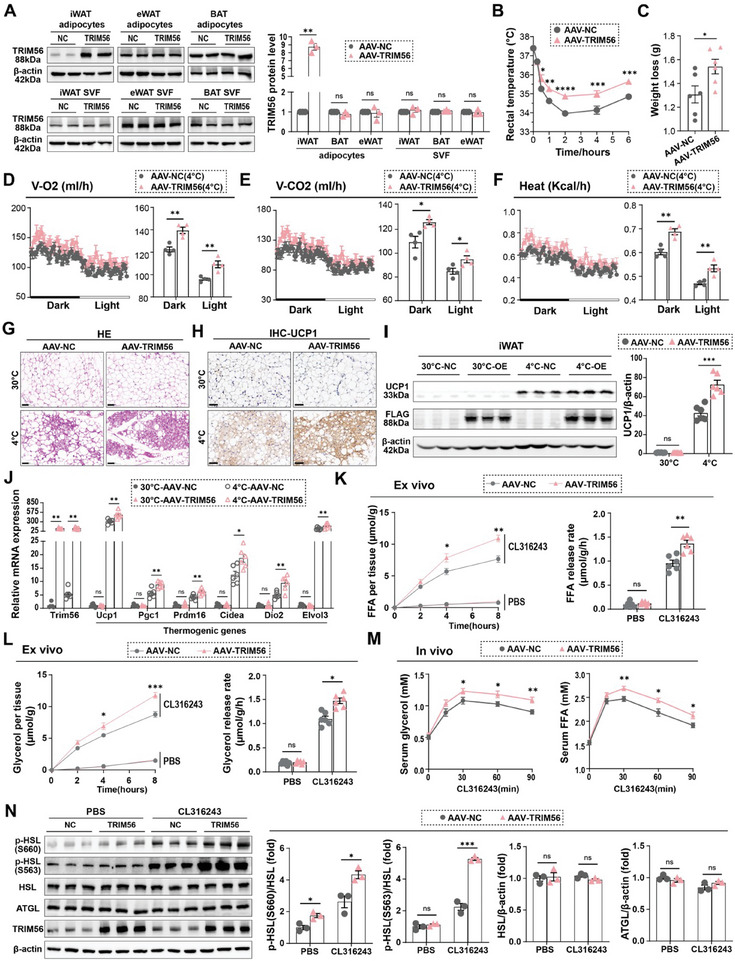
Increased TRIM56 expression promotes white adipose tissue browning. **A**) Immunoblot analysis was performed to assess TRIM56 protein in fractionated adipocytes and stromal vascular fraction (SVF) cells from interscapular BAT, iWAT and epididymal white adipose tissue (eWAT) of TRIM56‐overexpressing mice and control littermates. β‐actin protein was used as the loading control, with quantitative analysis shown on the right (*n* = 3). J) After being housed at 30 °C for two weeks, the mice were transferred to a 4 °C environment for three days. Assessments were performed at specific time points. B), Rectal temperature in the initial 6 h was recorded (*n* = 8). C) Weight loss in mice following 3 days of cold exposure (*n* = 6). D–F) Analysis of O2 consumption, CO2 production and energy expenditure (heat). The dark/light bar means a 12 h duration (*n* = 4). G), Represented HE staining images of iWAT from the mice housed in indicated environments. Scale bar, 50 um. H), Represented UCP1 immunostaining images. Scale bar, 50 um. I) Over‐expressed TRIM56 protein was tagged with FLAG. Represented immunoblotting analysis of UCP1, FLAG and β‐actin protein (left) with quantitative analysis (right, *n* = 6). J) RT‐qPCR analysis of TRIM56 and indicated thermogenic genes (*n* = 6). K,L) iWAT strips from TRIM56‐overexpressing mice and their control littermates were treated with PBS or cl316243 (1 µm) in vitro. The mice were normally housed at room temperature and were not subjected to cold exposure prior to the experiment. Released FFA (K) and glycerol (L) in medium were measured at specified time points (left), with release rates quantified (right, *n* = 6). L–N) Prior to the experiment, the mice were maintained at room temperature without exposure to cold. Four hours before intraperitoneal injection of CL316243, they were fasted. M), Serum glycerol and FFA levels in mice were monitored at various time points following intraperitoneal injection of CL316243 (1 mg kg^−^^1^, *n* = 8). N) Immunoblot analysis of HSL phosphorylation at Ser660 and Ser563 as well as ATGL protein in iWAT from mice treated with saline or CL316243 (1 mg kg^−1^, 4 h), with quantitative analysis shown on the right (*n* = 3). Data are presented as mean ± SEM. *, *P* < 0.05, **, *P* < 0.01, ***, *P* < 0.001, and ****, *P* < 0.0001. ns, non‐significant.

To examine the role of TRIM56 in adipocytes in vivo, we employed Adeno‐Associated Virus Serotype 9 (AAV9) vectors to achieve overexpression of the TRIM56 gene driven by the adiponectin promoter. These vectors were administered via subcutaneous injection into iWAT (Figure S3A, Supporting Information). This approach enabled targeted and augmented expression of TRIM56 exclusively within the adipocytes of iWAT. Subsequent Western blot and immunofluorescence analyses confirmed the successful overexpression of TRIM56 in the adipocytes of iWAT (**Figure** **3**A; Figure S3B,C, Supporting Information).

Mice injected with the specified AAV vectors were housed under thermoneutral conditions (30 °C) for a duration of 2 weeks. During this period, their metabolic rates were assessed using the Comprehensive Laboratory Animal Monitoring System (CLAMS). Under thermoneutral conditions, mice overexpressing TRIM56 exhibited comparable levels of CO2 production, O2 consumption, energy expenditure, locomotor activity, food intake, and respiratory exchange ratio (RER) when compared to those injected with the negative control AAV vectors (Figure S3D–F, Supporting Information).

Subsequently, mice were exposed to cold (4 °C), revealing that TRIM56 overexpression enhanced the ability to maintain core temperature (Figure 3B). Additionally, mice overexpressing TRIM56 exhibited increased CO2 production, O2 consumption, and energy expenditure (Figure 3D–F), with comparable locomotor activity, food intake, and RER between the groups (Figure S3H, Supporting Information). After three days of cold exposure, mice overexpressing TRIM56 lost more body weight, which is indicative of higher energy expenditure (Figure 3C). Histological examination using hematoxylin and eosin (HE) staining confirmed an increase in brown‐like adipocytes in the iWAT of TRIM56‐overexpressing mice, which was consistent with elevated levels of UCP1 protein (Figure 3G–I; Figure S3G, Supporting Information). Quantitative PCR analysis further revealed that TRIM56 significantly enhanced the expression of several key thermogenic genes, including *Prdm16, Dio2, Pgc1α, Cidea*, and *Elvol3*, as well as the expression of genes related to lipolysis and fatty acid oxidation, including *Pparα, Mcad, and Cpt1b* (Figure 3J; Figure S3K, Supporting Information). However, TRIM56 had no effect on the expression of adipogenesis‐related genes (Figure S3K, Supporting Information). No differences in the expression of thermogenic genes were observed in eWAT and BAT (Figure 3I,J, Supporting Information).

Given the pivotal role of lipolysis in activating adipocytes thermogenesis,^[^ ^27^, ^28^, ^29^, ^30^ ^]^ we investigated whether TRIM56 could regulate this process. In vitro lipolysis assays using iWAT explants demonstrated higher rates of glycerol and free fatty acid (FFA) release in mice overexpressing TRIM56 (Figure 3K,L). Consistently, in vivo lipolysis assays also showed that following injection with CL316243, serum levels of glycerol and FFA were significantly elevated, with an even greater increase observed in mice overexpressing TRIM56 compared to controls (Figure 3M). Further experiments confirmed that TRIM56 overexpression significantly enhanced the phosphorylation of HSL but had no effect on ATGL protein levels (Figure 3N). Collectively, these findings demonstrate that TRIM56 enhanced CL316243‐stimulated lipolysis in iWAT, which correlates with the promotion of adaptive thermogenesis through iWAT browning.

### 2.4 TRIM56 Depletion Inhibits Cold‐Induced iWAT Browning

To further explore the effects of TRIM56 depletion, we employed two methods to achieve TRIM56 knockout/knockdown (KO/KD) in adipocytes of iWAT, that is, locally injection of an AAV9 vector expressing Cre recombinase driven by the adiponectin promoter into TRIM56‐flox mice (TRIM56 KO), as well as locally injection of an AAV9 vector carrying short hairpin RNA targeting TRIM56 (shTRIM56) driven by the adiponectin promoter into WT mice (TRIM56 KD). Both approaches achieved a significant and specific reduction in TRIM56 protein levels in adipocytes of iWAT, as confirmed by Western blotting (Figures S4A and S5A, Supporting Information).

TRIM56 KO mice and their littermate controls were kept in a thermoneutral environment or underwent three days of cold exposure. During the initial 6 h of cold exposure, the rectal temperature of TRIM56 KO mice was lower than that of controls, suggesting impaired thermogenesis (Figure S4B, Supporting Information). Metabolic parameters showed no significant differences between the two groups in terms of activity, food intake, or RER (Figure S4F–H, Supporting Information). However, compared to the control group, TRIM56 KO mice exhibited reduced oxygen consumption, carbon dioxide production, and energy expenditure (Figure S4C–E, Supporting Information). Additionally, TRIM56 KO mice showed less body weight loss, which correlated with their lower metabolic rate (Figure S4I, Supporting Information). Further analysis of iWAT revealed that TRIM56 KO suppressed the expression of key thermogenic genes, including *Prdm16*, *Dio2*, *Pgc1α*, *Cidea*, and *Elvol3* (Figure S4J, Supporting Information). Histological analysis (HE and immunohistochemistry staining) and Western blot analysis showed that TRIM56 KO mice had reduced recruitment of beige adipocytes and significantly lower cold‐induced UCP1 protein expression (Figure S4K–M, Supporting Information).

As expected, TRIM56 KD mice exhibited the similar phenotype under the cold condition, that is, decreased energy expenditure, impaired cold tolerance, reduced beige‐like adipocytes within iWAT, as well as decreased UCP1 protein levels and thermogenic gene expression (Figure S5, Supporting Information). Collectively, these findings highlight the essential role of TRIM56 in cold‐induced iWAT browning and temperature maintenance in mice.

### 2.5 iWAT TRIM56 Overexpression Ameliorates Diet‐Induced Obesity and Metabolic Abnormalities

Next, we investigated the potential of TRIM56 to alleviate diet‐induced obesity by enhancing adaptive thermogenesis. Mice were injected with either AAV‐TRIM56 or AAV‐NC and subsequently fed either a high‐fat diet (HFD) or a standard control diet (SCD) for 12 weeks (Figure S6A, Supporting Information). During the SCD feeding period, food intake was comparable between the groups (Figure S6D, Supporting Information). Although mice overexpressing TRIM56 exhibited a trend of reduced body weight gain, the differences were not statistically significant (Figure S6B, Supporting Information). Similarly, no significant differences were observed in liver and adipose tissue weights (iWAT, eWAT, BAT), glucose tolerance, or insulin resistance across the groups (Figure S6C,F–H, Supporting Information). Morphological assessments of iWAT also showed no distinct differences (Figure S6E, Supporting Information).

**Figure 4:**
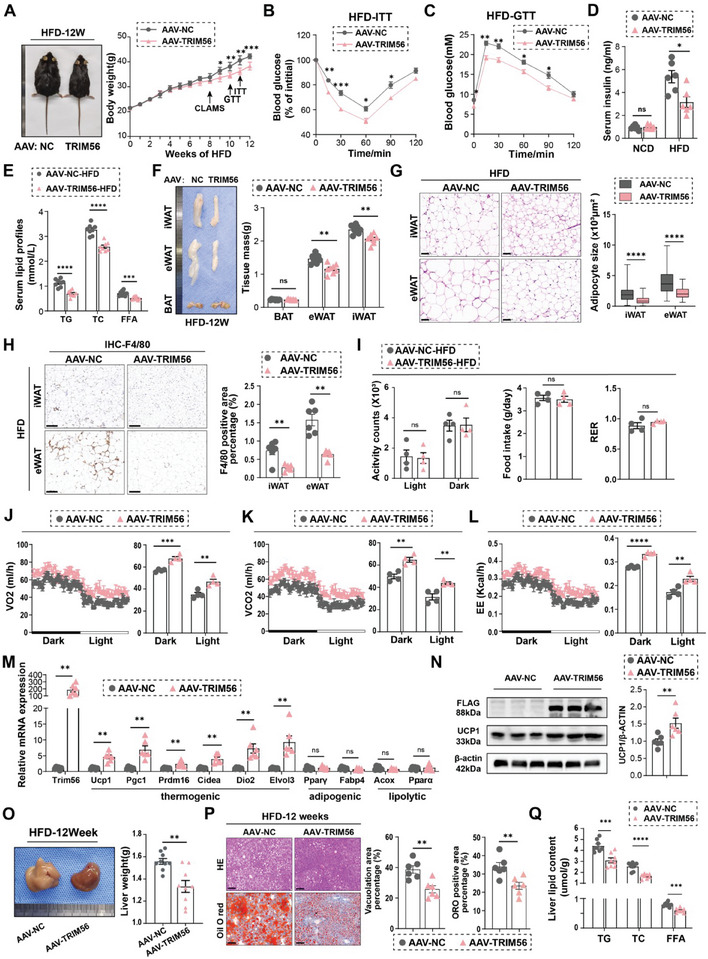
Enhanced TRIM56 expression in iWAT ameliorated diet‐induced obesity. Mice injected with AAV‐TRIM56 or AAV‐NC were fed a HFD for 12 weeks. Various assessments were conducted during this period to evaluate the impact of TRIM56 expression on metabolic parameters and adipose tissue characteristics. A) Represented gross morphology of mice fed with a 12‐weeks HFD (left) and the curve of body weight during the period of HFD (right, *n* = 8). B,C) Insulin tolerance test (B) and glucose tolerance test (C). *n* = 8. D) Fasting serum insulin from mice fed with a 12‐weeks of HFD and SCD (*n* = 6). E) Serum lipid contents (TG, TC, and FFA) from mice fed with a 12‐weeks of HFD (*n* = 8). TG, triglycerides. TC, total Cholesterol. FFA, free fat acid. F) Represented images of indicated tissues (iWAT, eWAT, BAT) from mice fed with 12‐weeks HFD (left) and quantitative analysis of tissues wights (right, *n* = 8). G) Represented HE staining images and adipocyte cell size measurements of indicated tissues (iWAT and eWAT). Right panel, the quantitative results of the size of each adipocyte counted (AAV‐NC‐iWAT: *n* = 101 cells; AAV‐TRIM56‐iWAT: *n* = 180 cells; AAV‐NC‐eWAT: *n* = 118 cells; AAV‐TRIM56‐iWAT: *n* = 176 cells). Scale bar, 50 um. H) Represented IHC staining images of F4/80 proteins of indicated tissues (iWAT and eWAT). Right panel, the quantitative results of the percentage of positive area (*n* = 6). Scale bar, 100 um. I) During the CLAMS‐monitored period, activity counts, food intake and respiratory exchange ratio (RER) were measured and shown. J–L) The O2 consumption, CO2 production, and energy expenditure (heat) of mice were analyzed (*n* = 4). The dark/light bar equals 12 h. M) RT‐qPCR analysis of TRIM56, key thermogenic markers, adipogenic markers and lipolytic markers (*n* = 6). N) Over‐expressed TRIM56 protein was tagged with FLAG. Represented immunoblotting images of UCP1, FLAG and β‐actin protein (left) with quantitative analysis (right, *n* = 6). O) Represented liver images(left) and quantitative analysis of liver wights (right, *n* = 8). P) Represented HE and Oil Red O‐stained liver section images. The quantitative results were shown in the right (*n* = 6). Q) Liver lipid contents (TG, TC, and FFA) were evaluated in mice after a 12‐week high‐fat diet regimen (*n* = 8). In addition to panels, I and J, each group had 6–8 mice. Data are presented as mean ± SEM. *, *P* < 0.05, **, *P* < 0.01, ***, *P* < 0.001, and ****, *P* < 0.0001. ns, non‐significant.

Under HFD conditions, mice overexpressing TRIM56 exhibited significantly less body weight gain compared to controls, despite comparable food intake (**Figure** **4**A; Figure S6I, Supporting Information). Furthermore, metabolic disturbances associated with obesity, such as glucose tolerance, insulin resistance, fasting serum insulin levels, and serum lipid levels, were all markedly improved in TRIM56‐overexpressing mice (Figure 4B–E).

Analysis of adipose tissue depots revealed decreased weights in both iWAT and eWAT in TRIM56‐overexpressing mice (Figure 4F). Consistent with these findings, histological examinations showed smaller adipocyte sizes in these mice (Figure 4G). It has been reported that obesity‐induced inflammatory status in WAT can lead to many pathogenic outcomes and impaired adaptive thermogenesis.^[^ ^31^ ^]^ Importantly, immunohistochemical analysis indicated reduced macrophage infiltration in the WAT of TRIM56‐overexpressing mice, which is associated with improved inflammatory status and enhanced adaptive thermogenesis (Figure 4H).

Given similar food intake between the two groups, we hypothesized that the protective effects of TRIM56 against obesity are mediated through elevated metabolic rates. Our CLAMS assessments revealed increased CO2 production, oxygen consumption, and heat production in AAV‐TRIM56 transgenic mice subjected to a high‐fat diet (Figure 4J–L). Activity levels, food intake, and RER during monitoring showed no discernible differences (Figure 4I). Furthermore, TRIM56 overexpression was found to upregulate the expression of thermogenic markers, including *UCP1, Prdm16, Dio2, Pgc1α, Cidea*, and *Elvol3*, whereas the expression of adipogenic and lipolytic markers remained unaffected (Figure 4M,N). In contrast, the expression of thermogenic genes remained unchanged in eWAT and BAT (Figure S6J–L, Supporting Information).

Ectopic lipid accumulation, a common consequence of obesity, frequently precipitates numerous metabolic dysfunctions such as hepatic steatosis.^[^ ^4^ ^]^ Examination of the livers from TRIM56‐overexpressing mice revealed a reduction in both liver weight and lipid content compared to controls (Figure 4O–Q). Collectively, our data demonstrate that overexpression of TRIM56 ameliorates diet‐induced obesity and associated metabolic abnormalities by potentiating adaptive thermogenesis.

### 2.6 TRIM56 Knockdown Aggravates Diet‐Induced Obesity

In further support of our findings, mice were subjected to a 12‐week HFD feeding after being injected with either AAV‐shTRIM56 or AAV‐shNC. Despite comparable food intake between groups, mice with TRIM56 knockdown exhibited greater weight gain compared to controls by the end of the 12‐week period (Figure S7A–C, Supporting Information). Conversely, while the overexpression of TRIM56 improved glucose tolerance and insulin sensitivity, its knockdown exacerbated these conditions (Figure S7D,E, Supporting Information). Additionally, serum insulin levels were also elevated in mice with TRIM56 knockdown (Figure S7F, Supporting Information).

Notably, a decrease in TRIM56 expression led to increased weights of both iWAT and eWAT, which was accompanied by an enlargement in adipocytes size as observed in HE staining (Figure S7G,I, Supporting Information). Metabolic assessments revealed significantly reductions in CO2 production, oxygen consumption, and heat production in AAV‐shTRIM56 mice compared to controls (Figure S7J,K, Supporting Information). However, locomotor activities, food intake, and RER remained comparable between the groups during this period (Figure S7H, Supporting Information).

In contrast to mice with TRIM56 overexpression, which showed increased expression of thermogenesis markers, mice with TRIM56 knockdown exhibited decreased expression of these markers (Figure S7L,M, Supporting Information). Moreover, TRIM56 knockdown also exacerbated the lipid accumulation in the liver of these obese mice (Figure S7N–P, Supporting Information). By integrating the findings of reduced TRIM56 expression under obesity conditions (Figure 1D–H), these results highlight the unique regulatory role of TRIM56 in the progression of obesity.

### 2.7 TRIM56 Modulates Adipocyte Thermogenesis by Interacting with TLE3

**Figure 5:**
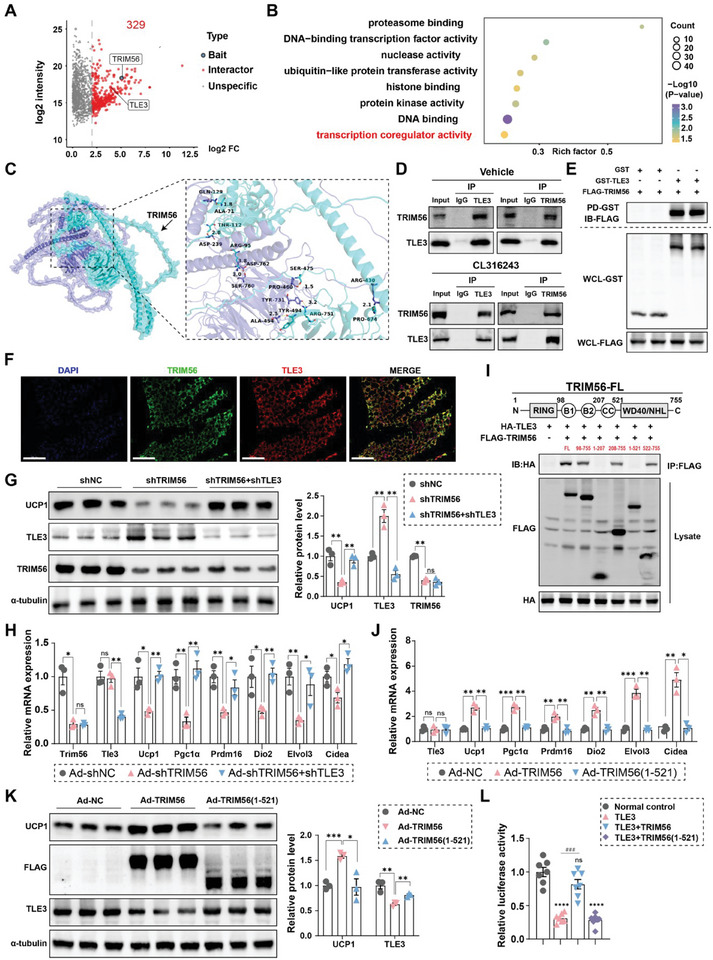
Identifies TLE3 as an interacting partner of TRIM56. A) Immunoprecipitation of TRIM56 was performed from SVF cell lysates that overexpressed TRIM56, followed by MS analysis. This approach identified a total of 329 interacting proteins. B) GO enrichment analysis was performed on the MS data. C) Molecular docking analysis indicated possible binding interactions between TRIM56 and TLE3 proteins. Turquoise, TRM56. Purple, TLE3. D) Immunoprecipitation confirmed the interaction between TRIM56 and TLE3 in adipose tissue, as detected in iWAT from mice treated with consecutive CL316243 injections for 5 days and PBS solvent injections. E) The purified FLAG‐TRIM56 protein was co‐incubated in vitro with either GST‐TLE3 or GST protein. A GST‐pulldown assay was used to confirm the interaction between TRIM56 and TLE3. WCL whole co‐incubated lysate. PD, pull down. F) Representative immunofluorescence staining of TLE3 and TRIM56 in iWAT was shown. TLE3 was labeled in red, TRIM56 in green, and DAPI in blue. Scale bars represent 100 µm. G,H) Adenoviral vectors carrying shRNA were used to knock down TLE3 and TRIM56 in SVFs. After 24 h of viral treatment, the cells were stimulated with 1 µm CL316243 for an additional 24 h. (G), Representative immunoblotting images of TRIM56, UCP1, TLE3 and α‐tubulin proteins (left), alongside their quantified intensities (right). (H), RT‐qPCR analysis of specified thermogenic markers. *n* = 3 per group. I) We created plasmids encoding FLAG‐tagged truncated fragments of TRIM56 and transfected them into HEK293T cells, which were also transfected with a plasmid encoding HA‐tagged TLE3. Immunoprecipitation was then performed to assess the interaction between TRIM56 fragments and TLE3. J,K) Adenoviral vectors were used to overexpressed FLAG‐tagged TRIM56 in mature adipocytes. After 24 h of viral treatment, the cells were stimulated with 1 µm CL316243 for an additional 24 h. (J), RT‐qPCR analysis of indicated thermogenic markers. (K), Representative immunoblotting images of FLAG, UCP1, TLE3 and α‐tubulin protein (left), alongside their quantified intensities (right). *n* = 3 per group. L) Fragments of the Ucp1 promoter linked to a luciferase reporter gene were transfected into HEK 293T cells alongside either control or TLE3‐overexpressing plasmids, with or without TRIM56‐expressing plasmids. The luciferase activity was then measured and normalized against the control. *n* = 7 per group. Data are expressed as mean ± SEM. *, *P* < 0.05, **, *P* < 0.01, ***, *P* < 0.001, and ****, *P* < 0.0001. ns, non‐significant.

To explore the mechanism by which TRIM56 promotes the browning process, we conducted immunoprecipitation of TRIM56 from SVF cells overexpressing TRIM56, followed by mass spectrometry (MS) analysis. We identified 329 proteins that interact with TRIM56 (**Figure** **5**A). Subsequent Gene Ontology (GO) enrichment analysis revealed significant enrichment in biological processes including proteasome binding, DNA‐binding transcription factor activity, and nuclease and transcription coregulator activity (Figure 5B). These findings suggest that TRIM56 plays a role in transcriptional regulation and post‐transcriptional modifications. Notably, among the interacting proteins, TLE3 stood out due to its established function as a negative regulator of browning processes.^[^ ^32^, ^33^ ^]^

Molecular docking analysis suggested potential binding interactions between TRIM56 and TLE3 proteins (Figure 5C). Furthermore, we confirmed their interaction using co‐immunoprecipitation (Co‐IP) under both basal conditions and CL316243 stimulation (Figure 5D). Subsequently, a GST‐pulldown assay was conducted to ascertain the interaction between TRIM56 and TLE3 was direct and not mediated by intermediary proteins (Figure 5E). Additionally, immunofluorescence staining provided visual evidence of the co‐localization of TRIM56 and TLE3 within adipose tissues (Figure 5F).

Next, we investigated whether TRIM56's effect on browning requires TLE3. By silencing TLE3 in SVF cells, we observed a reversal of the suppression in thermogenesis gene expression that was induced by TRIM56 knockdown (Figure 5G,H). Using truncated fragments of TRIM56, we pinpointed the specific region, amino acids 522 to 755, was responsible for its interaction with TLE3, as evidenced by the absence of interaction with a truncated fragment encompassing amino acids 1 to 521 (Figure 5I). Functional assays further demonstrated that deletion of amino acids 522–755 compromised TRIM56's ability to enhance thermogenesis gene expression in SVF cells (Figure 5J,K).

Finally, dual‐luciferase reporter assays showed that TRIM56 counteracts the inhibitory effect of TLE3 on the UCP1 promoter (Figure 5L). These findings highlight the importance of TLE3 in TRIM56‐mediated regulation of the browning process.

### 2.8 TRIM56 Facilitates the Degradation of TLE3

**Figure 6:**
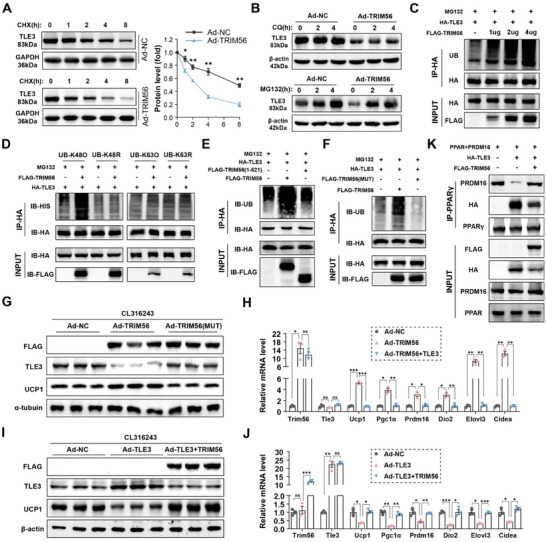
TRIM56 promotes TLE3 degradation. A) Adenoviral vectors were used to overexpressed FLAG‐tagged TRIM56 in mature adipocytes. WB analysis conducted on TLE3 and GAPDH in SVFs treated with cycloheximide (CHX) for various time periods, alongside their quantified intensities (*n* = 3). B) SVFs were treated with control or FLAG‐TRIM56 adenoviral vectors, alongside MG132 or CQ. TLE3 and β‐actin protein expression levels were measured at specified time points. C) HEK293T cells were transfected with varying concentrations of FLAG‐TRIM56 and HA‐TLE3 plasmids, with the treatment of MG132. Then, IP was conducted. D) HEK293T cells were transfected with HA‐TLE3, FLAG‐TRIM56, and HIS‐tagged ubiquitin variants (active K48‐linked form K48O, inactive K48‐linked mutant K48R, active K63‐linked form K63O, inactive K63‐linked mutant K63R) in the presence of MG132. Subsequently, IP was conducted. E) In the presence of MG132, HEK293T cells were co‐transfected with HA‐TLE3 along with ubiquitin (UB), and also with FLAG‐tagged TRIM56 variants: the full length TRIM56 or the truncated fragment spanning amino acids 1 to 521. Then, IP was conducted. F) HEK293T cells were co‐transfected with HA‐TLE3 along with ubiquitin (UB), and also with FLAG‐tagged TRIM56 variants. These included the wild‐type TRIM56 and a mutant variant (21AACC24) that lacks E3 ubiquitin ligase activity. Then, IP was conducted. G,H) Adenoviral vectors were used to overexpressed FLAG‐tagged TRIM56 variants in mature adipocytes. After 24 h of viral treatment, the cells were stimulated with 1µM CL316243 for an additional 24 h. G), Representative immunoblotting images of FLAG, UCP1, TLE3 and α‐tubulin proteins. H), RT‐qPCR analysis of indicated thermogenic markers (*n* = 3). I,J) Adenoviral vectors were used to overexpress TLE3 and FLAG‐tagged TRIM56 in mature adipocytes. After 24 h of viral treatment, the cells were stimulated with 1 µm CL316243 for an additional 24 h. I), Representative immunoblotting images of FLAG, UCP1, TLE3 and β‐actin proteins. J), RT‐qPCR analysis of specified thermogenic markers (*n* = 3). Data are presented as mean ± SEM. *, *P* < 0.05, **, *P* < 0.01, ***, *P* < 0.001, and ****, *P* < 0.0001. ns, non‐significant.

In our study, we observed increased expression of TLE3 in subcutaneous adipose tissue samples obtained from obese individuals with a BMI exceeding 30 (Figure S8I,J, Supporting Information). Consistently, in mice, TLE3 expression levels were elevated in subcutaneous adipose tissue following a high‐fat diet, whereas these levels decreased under cold conditions (Figure S8A–D,E–H, Supporting Information). Notably, we found a negative correlation between the protein levels of TRIM56 and TLE3 in both mice and humans (Figure S8B,D,F,H, Supporting Information), despite comparable mRNA levels for both proteins (Figure S8A,C,E,G, Supporting Information). Based on these findings, we hypothesized that TRIM56 facilitates the degradation of TLE3. To investigate this, we conducted experiments where overexpression of TRIM56 in the presence of cycloheximide (CHX), a protein synthesis inhibitor, resulted in a reduction in the stability of the TLE3 protein (**Figure** **6**A). This reduction in stability was reversed upon the using of MG132 (a proteasome inhibitor) but not by CQ (chloroquine), suggesting that TRIM56‐mediated regulation of TLE3 is proteasome dependent (Figure 6B).

Since proteins targeted for proteasome‐mediated degradation typically feature K48‐linked ubiquitination,^[^ ^22^, ^34^, ^35^, ^36^ ^]^ our focus was on determining whether TRIM56 promotes the ubiquitination of TLE3. Cells transfected with TRIM56‐overexpressing plasmids exhibited a significant, dose‐dependent increase in TLE3 ubiquitination compared to those transfected with empty vectors (Figure 6C). Further investigation confirmed that this ubiquitination was indeed K48‐linked, as evidenced by elevated levels of TLE3 ubiquitination in cells overexpressing the active K48‐linked ubiquitin (K48O) plasmids, but not the inactive K48‐linked mutant ubiquitin (K48R) or the K63‐linked ubiquitin (K63O) (Figure 6D).

To further validate our findings, we employed two different TRIM56 mutants. Deletion of the C‐terminal domain (522‐755 aa) of TRIM56 abolished its interaction with TLE3. Additionally, overexpression of the N‐terminal fragment of TRIM56 (1‐521 aa) failed to augment TLE3 polyubiquitination, highlighting the necessity of the C‐terminal domain for TRIM56‐TLE3 interaction and TLE3 protein instability (Figure 6E). Furthermore, the TRIM56 variant (21AACC24), which has been previously documented to lacks E3 ubiquitin ligase activity,^[^ ^22^, ^37^ ^]^ was unable to affect the ubiquitination and stability of TLE3 (Figure 6F). Overexpression of this variant in SVF cells does not recapitulate the role of wild‐type TRIM56 in promoting the browning process (Figure 6G,H).

Subsequently, we investigated whether TRIM56 could counteract the function of TLE3. Previous studies have suggested that TLE3 competes with PRDM16 to bind to PPARγ, a process that inhibits the expression of genes involved in heat production.^[^ ^32^, ^33^ ^]^ Our findings confirmed that an elevation in TRIM56 levels enhanced the binding of PRDM16 to PPARγ (Figure 6K). When SVF cells were stimulated with CL316243, overexpression of TLE3 effectively suppressed the expression of key thermogenic genes. However, the suppressive effect was counteracted by the overexpression of TRIM56 (Figure 6I,J). These findings indicate that TRIM56 enhances adipocyte thermogenesis by facilitating the degradation of TLE3.

### 2.9 Increased Expression of TLE3 Negates the Effects of TRIM56 on Adaptive Thermogenesis

**Figure 7:**
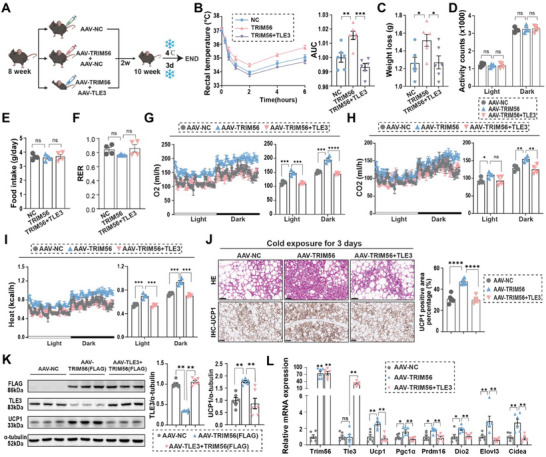
TLE3 overexpression disrupts the enhancement effect of TRIM56 on adaptive thermogenesis. A) Experimental design schematic: 8‐week‐old mice were injected with AAV to over‐express TRIM56 or TLE3. Following a 2‐week maintenance at 30 °C, they were subjected to a 3‐day exposure at 4 °C. This figure was created with BioRender.com/126k237. B) Mice rectal temperatures were recorded at specified intervals following the onset of cold exposure, and their quantification was also noted (*n* = 6). C) Assessment of weight loss following a 3‐day cold challenge (*n* = 6). D–F) During the CLAMS‐monitored period, D) activity counts, E) food intake and F) respiratory exchange ratio were measured and shown. G–I) The O2 consumption, CO2 production and energy expenditure of mice subjected to cold exposure were analyzed, along with their quantitative assessment (*n* = 4). The dark/light bar equals 12 h. J) Representative HE and UCP1 IHC staining images of iWAT. Right panel, the quantitative results of the percentage of UCP1 positive area (*n* = 6). Scale bar, 50 µm. K) Representative immunoblotting images of FLAG, UCP1, TLE3, and α‐tubulin proteins, alongside their quantified intensities (*n* = 6). I) RT‐qPCR analysis of key thermogenic markers (*n* = 6). Data are expressed as mean ± SEM. Each group had 6–8 mice. *, *P* < 0.05, **, *P* < 0.01, ***, *P* < 0.001, and ****, *P* < 0.0001. ns, non‐significant.

To confirm the importance of the TRIM56‐TLE3 pathway in regulating the browning processes, we employed AAV vectors to increase the expression of TRIM56 and TLE3 specifically in the adipocytes of iWAT (**Figure** **7**A). Following a two‐week interval after the AAV injection, mice were subjecte to three days of cold exposure. Our results demonstrate that the beneficial effects of TRIM56 overexpression on thermogenesis, including improved maintenance of core body temperature and increased weight loss after cold exposure, were compromised by enforcing TLE3 expression (Figure 7B,C). The CLAMS analysis provided direct evidence that overexpression of TLE3 reversed the TRIM56‐mediated increases in CO2 production, O2 consumption, and energy expenditure (Figure 7G–I). During this period, activity counts, food intake, and RER were comparable between each group (Figure 7D–F).

Moreover, our investigation revealed that TLE3 overexpression attenuated the TRIM56‐induced upregulation of thermogenic genes (Figure 7K,L). Histological examination and immunohistochemical analysis further demonstrated that the TRIM56‐mediated recruitment of beige adipocytes was suppressed by TLE3 overexpression (Figure 7J). These results confirmed the regulatory function of the TRIM56‐TLE3 axis in adipose tissue browning and its implications for metabolic health.

## 3 Discussion

Obesity imposes a formidable health burden on billions of individuals worldwide. Increasingly, research is focusing on combating obesity by enhancing the function of thermogenic adipocytes, including both beige and brown types. A direct and efficient strategy to address this issue lies in regulating protein stability. Our study first uncovered the pivotal role of the TRIM56‐TLE3 axis in orchestrating the browning process of white adipose tissue. Our findings reveal that TRIM56 expression is upregulated in response to cold exposure but downregulated during obesity. Mechanistically, TRIM56 promoted the expression of thermogenic genes by facilitating the ubiquitination and subsequent degradation of TLE3. Based on this mechanism, we considered that augmenting TRIM56 levels in adipocytes could offer a promising therapeutic approach to ameliorate diet‐induced obesity and associated metabolic disorders.

TLE3, a member of the Groucho/Transducin‐like enhancer of split (TLE) family, plays a pivotal role in determining cellular identity. Despite the fact that members of this family do not bind to DNA directly,^[^ ^38^, ^39^ ^]^ they regulate transcription by interacting with various partner proteins, including members of the FOX family, HES, and TCF/LEF.^[^ ^40^, ^41^, ^42^, ^43^, ^44^ ^]^

TLE3 negatively regulates brown‐selective genes and inhibits the differentiation of beige adipocytes.^[^ ^32^, ^33^, ^44^, ^45^ ^]^ Research has demonstrated that TLE3 regulates gene expression in adipocytes by binding to PPARγ and EBF2, thereby enabling it to adapt to the body's fluctuating metabolic demands.^[^ ^32^, ^33^ ^]^ An increase in TLE3 protein levels leads to an upregulation of genes involved in lipid accumulations and a downregulation of those associated with adaptive thermogenesis. Additionally, the inactivatiion of TLE3 has been shown to ameliorate the decline in thermogenic capacity associated with adipose tissue aging.^[^ ^32^, ^33^ ^]^ These findings align with our observations and provide supporting evidence that the TRIM56‐TLE3 pathway plays a crucial role in regulating thermogenesis in adipocytes. Notably, we observed increased mRNA expression and protein levels of TLE3 in both adipose tissues of obese mice and humans. However, either TRIM56 overexpressing or KD only regulates TLE3 protein levels without affecting TLE3 gene expression. We believe that the elevation in TLE3 protein levels in obesity subjects is partly due to an increase at the transcriptional level, as well as a reduction in protein degradation caused by decreased TRIM56 levels. Although enforcing TRIM56 expression does not affect TLE3 mRNA levels, it effectively degrades TLE3 protein and inhibits its function. However, the mechanism of TLE3 transcriptional regulation in the obese condition needs further exploration.

TLE3's regulatory function extends beyond the adipose tissue. By interacting with various partner proteins, TLE3 influences a wide range of transcriptional processes throughout the body. Numerous studies have underscored its pivotal role in the onset and progression of tumors. Specifically, TLE3 has been implicated in inhibiting breast cancer cell migration,^[^ ^39^ ^]^ suppressing the progression of colorectal cancer,^[^ ^46^ ^]^ and regulating the differentiation of rhabdomyosarcoma cells.^[^ ^47^ ^]^ Recent studies have also brought attention to TLE3's emerging function in immune regulation. Earlier research demonstrated that the transcription factor Hhex, in collaboration with TLE3, promotes the development of memory B cells.^[^ ^48^ ^]^ More recent findings suggest that targeting TLE3 can reprogram antigen‐experienced CD8 T cells, facilitating the generation of central memory T cells (TCM).^[^ ^45^ ^]^ In our present study, we could clearly see, more convinced in eWAT, decreased abundance of macrophages and crown‐like structures (CLS), which relates largely to the chornic inflammation status of WAT, in TRIM56‐overexpressed mice. We believe this may be secondary to the reduction in adipose tissue weight or the improvement in overall metabolic status, since local injection of AAV vectors containing the adiponectin promoter into iWAT did not affect the level of TRIM56 in other adipose pads, nor did in other cell types. Of course, we think that TRIM56‐TLE3 axis in macrophages may also participate in the thermogenesis and obesity by regulating macrophage behavior, which is also a very worthwhile direction for exploration.

As to the the noticeable reduction in eWAT, we believe it to be secondary to the activation of iWAT browning. It is reasonable since iWAT browning increases thermogenesis and energy expenditure, indirectly promoting the lipolysis of eWAT and whole‐body energy metabolism. Although we did not examine other WAT, such as omental adipose tissue and perirenal adipose tissue, we believe that there is a strong likelihood that their functions, just like those of eWAT, have also been improved.

Despite the promising potential for clinical applications, no agonists or inhibitors targeting TLE proteins have been identified so far. In this context, our study uncovers a novel mechanism that regulates TLE3 protein stability, potentially opening new avenues for modulating adipose tissue function. Notably, we highlight the importance of the 522–755 amino acid segment of TRIM56 in recognizing TLE3. Interestingly, this segment is also indispensable for binding to FASN, a process that contributes to alleviating MAFLD.^[^ ^22^ ^]^ Targeting this amino acid segment with small molecules may offer therapeutic potential for a wide range of metabolic diseases.

While there are numerous medical interventions available for weight reduction, many come with potential side effects. Treatment targeting TRIM56‐TLE3 axis holds promise as a novel therapeutic strategy for obesity and related metabolic disorders. Despite its involvement in multiple physiological processes and disease states, TRIM56, similar to TLE3, remains largely unexplored as a therapeutic target. Its untapped potential for medical applications underscores the importance of future research and drug development efforts focused on this protein.

## 4 Experimental Section

### Animal Studies

Animal experiments conducted in this study were approved by the Tongji Medical College Ethics Committee, Huazhong University of Science and Technology. Mice were procured from Weitonglihua Co. Ltd. (Beijing, China) and housed under pathogen‐free conditions with a 12 h light/12 h dark cycle, at a humidity of 40%–60% and a temperature of 23 °C. They had ad libitum access to food and water throughout the experiments.

Adiponectin‐promoter‐containing AAV9 vectors carrying either the TRIM56 gene was employed for overexpression, or short hairpin RNA targeting TRIM56 (shTRIM56) for knockdown specifically in inguinal white adipose tissue (iWAT). Control groups received vectors carrying scramble shRNA or negative controls. AAV9 vectors were subcutaneously injected in eight points on each side of the iWAT.

In the case of aged mice, injections were administered at 18 months of age. Additionally, injections were given to younger mice. Following injection, mice were housed either at room temperature or at 30 °C for two weeks to ensure stable expression of the AAV vectors.

To induce diet‐induced obesity, mice injected with AAV9 were fed a high‐fat diet (Research Diet, D12492, 60% kcal fat) or a standard control diet (SCD) for three months. Weekly monitoring was conducted for bodyweight and food intake. For CL316243 stimulation, mice received intraperitoneal injections of CL316243 at 1 mg kg^−1^ daily for five consecutive days. Cold challenges were conducted by exposing mice to 4 °C for three days.

Prior to sacrifice, mice were anesthetized, and blood serum as well as specified tissues were collected for further analyses.

### Virus Preparation

HANBIO Co. Ltd. (Shanghai, China) provided all adenoviruses and adeno‐associated viruses (AAV). Each mouse received an injection of AAV at a dosage of 1 × 10^11 viral particles per side of inguinal white adipose tissue (iWAT). These vectors were utilized to manipulate the expression of specific genes within the adipose tissue of the mice.

### Isolation of SVFs and Differentiation to Mature Beige Adipocytes In Vitro

SVFs were isolated from the iWAT of 6‐week‐old mice following a previously established protocol.^[^ ^49^, ^50^, ^51^ ^]^ In brief, adipose tissue samples were enzymatically digested using collagenase I. The resulting SVFs were cultured in DMEM medium supplemented with 10% fetal bovine serum and 1% penicillin/streptomycin.

Upon reaching confluence after 48 h, the cultured medium was supplemented with specific differentiation agents: dexamethasone (2 µm), 3‐isobutyl‐1‐methylxanthine (0.5 mm), insulin(10 µg mL^−1^), and rosiglitazone (2.5 µm). After another 48 h, dexamethasone and 3‐isobutyl‐1‐methylxanthine were removed, while insulin and rosiglitazone were retained. Cells were cultured for 6–8 days until mature adipocytes were obtained for subsequent experiments.

### Fractionation of Adipocytes

The isolation of adipocytes was performed following protocols outlined in previous literature.^[^ ^26^, ^50^, ^52^ ^]^ Briefly, the procedure was similar to that used for extracting SVF components. Adipose tissue was minced and digested for 1 h, after which the upper layer of adipocyte suspension was collected by centrifugation. The adipocyte fraction was then washed three times with PBS and cultured in DMEM medium supplemented with 10% fetal bovine serum and 1% penicillin/streptomycin. After 6 h, the cells were ready for experimental procedures.

### Blood Glucose Measurements

For GTT, mice were fasted for 16 h prior to receiving an intraperitoneal glucose injection (2 g kg^−1^, Sigma‐Aldrich, D9434). This test assesses how efficiently the mice process glucose after a period of fasting, providing insight into their metabolic response to a glucose load.

Similarly, for ITT, mice were fasted for 4 h before receiving an intraperitoneal insulin injection (0.75 units kg^−1^, Sigma‐Aldrich, 1342106). Blood glucose levels were measured at indicated time points.

### Metabolic Analysis

The metabolic analysis was performed using the Comprehensive Lab Animal Monitoring System (CLAMS, Columbus Instruments) was used to measure metabolic rates under a 30 °C or 4 °C conditions for 24 h (12 h light/dark cycle). Prior to measurement, the mice will undergo a 3‐day acclimation period, and a computer would automatically control the room temperature. It was free access to food and water for the mice.

### Lipid and Insulin Analyses

Commercial assay kits were used to measure lipid contents in serum and liver (TG‐S0219S, TC‐S0211 M, FFA‐S0215S, all kits were purchased from Beyotime). ELISA kit (EZRMI‐32313K, Millipore) was used to measure serum insulin levels. All operations were conducted as per the manufacturer's instructions.

### Plasmids

The plasmids utilized in this study were sourced from MiaolingBio (Wuhan, China). The mutated variants of TRIM56 (Q9BRZ2) were created by MiaolingBio and subsequently verified through gene sequencing conducted by Tsingke Biotechnology Co., Ltd. (Wuhan, China).

### Molecular Docking Analysis

The structures of TLE3 and TRIM56 were predicted using Alphafold. The protein sequences of TRIM56 and TLE3 were displayed in Table S4 (Supporting Information). To validate the docking results, the protein preparation was carried out using AutoDockTools‐1.5.7, where water molecules were manually removed, and polar hydrogens were added. Protein‐protein docking was performed using the GRAMM Docking Web Server.^[^ ^53^ ^]^ The resulting protein‐protein complex was further refined by manually removing water molecules and adding polar hydrogens using AutoDockTools‐1.5.7. Finally, the protein‐protein interactions were predicted, and a visualization of the complex was created using PyMOL. In the figure, the TLE3 protein was depicted as a slate‐colored cartoon, TRIM56 as a cyan‐colored cartoon, with their respective binding sites represented as colored stick models. When focusing on the binding region, the corresponding protein is highlighted to show the binding site specific to it.

### Production of TRIM56 cKO Mouse Model

TRIM56‐flox mice (Strain S‐CKO‐11074) were purchased from Cyagen (China, suzhou). For the TRIM56 conditional knockout (cKO) mouse, exons 1 to 3 were selected as the cKO region. To construct the targeting vector, homologous arms and the cKO region were generated by PCR using the BAC clone RP24‐359E6 as a template. The gRNAs targeting the TRIM56 gene (gRNA‐A1:CCTGCTTTTCCGCTGGCAAGAGG;gRNA‐A2:CAGCTAAAGCGGCTTTTGGCTGG), along with a donor vector containing loxP sites and Cas9 mRNA, were co‐injected into fertilized mouse eggs to generate TRIM56 cKO offspring. F0 founder animals were identified by PCR followed by sequencing, and these founders were bred to wild‐type mice to assess germline transmission and produce F1 generation animals. The homozygous animals were injected locally with adiponectin‐driven AAV virus to express Cre protein, thereby achieving iWAT‐specific knockout.

### Recombinant Protein Expression and Purification

The recombinant plasmids for human TRIM56 and TLE3 were synthesized by MiaolingBio (Wuhan, China). The plasmid containing FLAG‐tagged TRIM56 was transfected into HEK293T cells. FLAG‐TRIM56 protein was then purified using a commercial kit (P2181 m, Beyotime, China). For the purification of GST‐tagged TLE3, the process followed established protocols.^[^ ^54^, ^55^ ^]^ Briefly, the corresponding recombinant plasmid was transfected into specialized *E. coli* strain for expression. Afterward, the bacteria were resuspended in lysis buffer (50 mm Tris‐HCl, pH 7.5, 100 mm NaCl, 1 mm DTT, 0.2 mm PMSF, and complete EDTA‐free protease inhibitor) and subjected to sonication on ice. Glutathione agarose (50‐80 µl per milliliter of protein lysate) was then added, and the mixture was incubated overnight at 4 °C with gentle agitation. The solution was washed six times with PBS, followed by elution with elution buffer (50 mm Tris‐HCl, pH 8, 100 mm NaCl, 10 mm reduced glutathione, 1 mm DTT). After a final 10 min wash with the elution buffer, low‐speed centrifugation was performed to collect the supernatant, resulting in purified GST‐TLE3 protein. The purified TRIM56 and TLE3 proteins were used for in vitro co‐incubation followed by immunoprecipitation to verify their interaction.

### Co‐Immunoprecipitation (co‐IP) Assay

To collect proteins from tissues and cells, use a lysis buffer that did not contain SDS. Combine the protein lysate with either the specific antibody or an IgG control antibody, and incubate overnight on a rotating shaker at 4 °C. Following this, add Protein A/G magnetic beads and incubate for an additional 4 h. Wash the mixture six times with a washing buffer, then add 5× loading buffer and heat at 100 °C for 10 min. The formulation for the washing buffer was based on published literature.^[^ ^55^ ^]^

### Ubiquitination Assay

Cells were exposed to 20 µm MG132 for 6 h before collection. Post‐treatment, the cells were lysed and heated for 5 min in IP buffer containing 1% SDS. The resulting lysate was diluted with IP buffer to a final concentration of 0.1% SDS. The supernatant was harvested following centrifugation at 12000 × g for 10 min. The standard co‐IP protocol was followed.

### MS

SVF cells were infected with adenoviruses expressing Flag‐TRIM56 or FLAG (normal control) for 24 h, then treated with 20 µm MG132 for 4 h. Cell lysates were processed using immunoprecipitation with Flag tag‐specific antibodies, and MS was employed to identify interacting proteins with TRIM56.

### LC‐MS/MS Analysis

MS analysis was performed using the timsTOF Pro system from Bruker. The resulting data were processed with MaxQuant software, employing Andromeda as the search algorithm. Proteins exhibiting a fold change greater than 4 between experimental and control samples were identified as potential interaction partners of the bait protein. To determine the biological relevance of these interactions, enrichment analysis was conducted using a two‐tailed hypergeometric test. GO terms with a P‐value below 0.05 were deemed significantly enriched among the interacting proteins.

### Western Blot

Tissue and cell lysates were collected using RIPA lysis buffer (G2002, Servicebio, Wuhan, China). After sonication on ice, samples were centrifuged at 12,000 g for 15 min to obtain clear lysates. Protein concentration was measured using the BCA kit (G2026, Servicebio, Wuhan, China). Equal proteins were loaded onto 8%–10% SDS‐PAGE gels and transferred onto PVDF membranes. Blocking was performed with 5% skimmed milk or BSA in TBST at room temperature for 2 h. PVDF membranes were then incubated overnight at 4 °C with primary antibodies, followed by secondary antibody incubation. Chemiluminescent signals were detected using a Clinx imaging system. The antibodies used in this study were listed in Table S2 (Supporting Information).

### RNA Extraction and Gene Expression Analysis

RNA was extracted from samples using TRIZOL reagent (Invitrogen, 15 596 018). For cDNA synthesis, commercial reagents from Takara (RR036 and RR086) were utilized. Gene expression was quantified with the ABI 7500 Real‐Time PCR system (Thermo Fisher). Sequence of primers used in this study was listed in Table S3 (Supporting Information).

### Seahorse

SVF cells were plated in XF24 cell culture microplates (Agilent, 100777‐004). Approximately 10000 cells per well were differentiated over 8 days. The mature adipocytes were washed twice with XF24 medium (Agilent, 103575‐100). After a 1 h pre‐incubation in XF24 medium at 37 °C without CO2, OCR was measured using the XF Extracellular Analyzer (Agilent, 102340‐100). Reagents were sequentially introduced into the XF24 wells at the specified times after being preloaded in cartridges (4µM norepinephrine, 1.5 µm oligomycin, 1.5 µm FCCP, 2 µm rotenone, and 2 µm Ant A).

### Transmission Electron Microscopy

Tissues were freshly sampled from designated areas, with each sample taken within 1–3 min and measuring ≈1–3 mm^3^. The small tissue blocks were transferred to EP tubes with fresh electron microscopy fixative for continued fixation at 4 °C. Samples were sectioned into 60–70 nm slices using an ultramicrotome, stained, and examined under a Hitachi HT7800 transmission electron microscope. A collection of images was collected for analysis.

### Immunofluorescence Staining

In this study, immunofluorescence was conducted on sections of iWAT. For the double staining procedure, tissue sections were incubated overnight at 4 °C with a combination of anti‐TRIM56 antibody and either anti‐TLE3, anti‐Adiponectin, or anti‐FLAG antibody. The sections were then incubated for 30 min with secondary antibodies conjugated to AlexaFluor488 (green) and AlexaFluor568 (red). The detection of antigens was visualized using a fluorescence microscope and analyzed with Axiovision4.8 software (Olympus, Japan, Tokyo).

### Histological Analysis

Adipose and liver specimens were immersed in formalin solution and subsequently embedded in paraffin. The resulting sections were then stained using H&E and immunofluorescence methods. For Oil Red O staining, the specimens were rapidly frozen, sectioned using a cryostat, and then stained.

### Luciferase Reporter Assay

The UCP1 promoter was cloned from human genomic DNA into the pGL3.0‐basic vector (Promega, E1751) using an In‐Fusion HD cloning kit (Takara, 639 648). The resulting plasmids were co‐transfected into HEK293T cells. After 24 h, luciferase activity in the cell lysates was measured using the dual‐luciferase assay system (RG027, Beyotime, China).

The primers used for amplifying the promoter were detailed in Table S1 (Supporting Information).

### Human Participants

Subcutaneous adipose tissue samples were obtained from patients at Union Hospital, Tongji Medical College, and Huazhong University of Science and Technology. All patients provided informed consent. This study was ethically approved.

### Statistics

Data were expressed as mean ± s.e.m. Statistical analyses were performed using an unpaired two‐tailed Student's t‐test, one‐way ANOVA, or two‐way ANOVA with Bonferroni's posttest in GraphPad Prism v.9.0.

### Ethical Approval Statement

The use of volunteer samples was approved by the Ethics Committee of Tongji Medical College, Huazhong University of Science and Technology (approval no. S092). All animal experiments were approved by the Laboratory Animal Center of Huazhong University of Science and Technology (approval no. 4386). It was ensured that all participants provided written informed consent and retained the right to withdraw from the study at any time; data collection and processing adhere to privacy protection principles, with all personally identifiable information de‐identified or removed.

## Conflict of Interest

The authors declare no conflict of interest.

## Author Contributions

H.Q. and Y.Z. contributed equally to this work. K.H. and M.D. conceived and designed the studies. H.Q. and Y.Z. performed most of the experiments and analyzed the data. J.H and Y.M. provided essential reagents and assisted with the experimental design and data analysis. H.Q. and Y.Z. wrote the manuscript.

## Supporting information



Supporting Information

## Data Availability

The data that support the findings of this study are available in the supplementary material of this article.
