# Supplementary material for: TRIM56 Promotes White Adipose Tissue Browning to Attenuate Obesity by Degrading TLE3
Source: Adv Sci (Weinh). 2025 Feb 10;12(13):2414073. doi: 10.1002/advs.202414073 (PMC11967773; doi:10.1002/advs.202414073)
Supplement: Supplementary file 1 — Supporting Information [file ADVS-12-2414073-s001.docx]

**Extended Data**

**
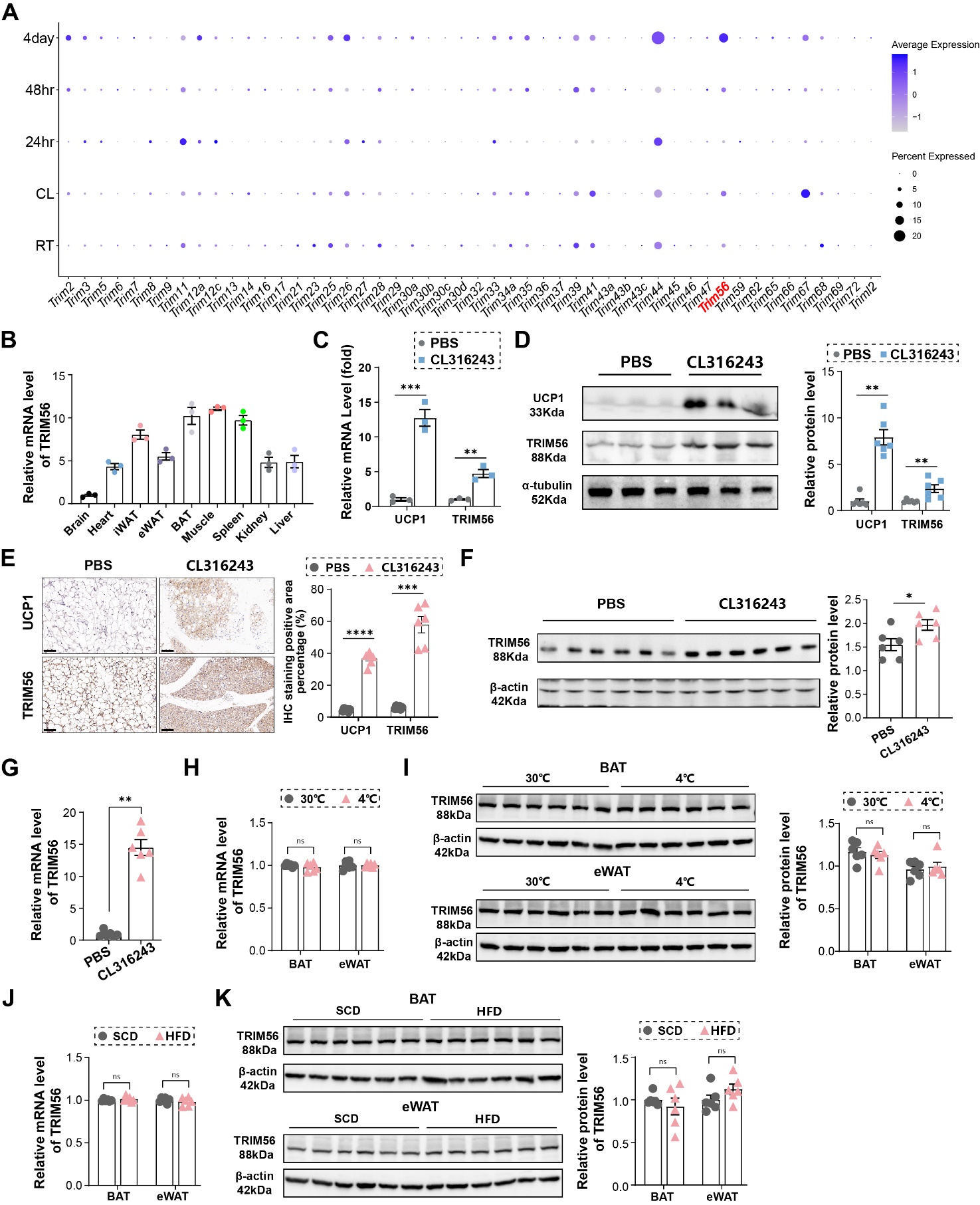
Extended Fig.1. TRIM56 expression in adipocytes was increased in response to CL316243 stimulation.**

**A**, The expression changes of TRIM family members in adipocytes of iWAT from mice subjected to cold exposure and CL316243 treatment were analyzed using the single-cell RNA sequencing dataset (GSE133486).

**B**, RT-qPCR analysis of TRIM56 mRNA levels in indicated tissues from C57BL/6J mice (n=3).

1. **D**, SVF cells were treated with 1μM CL316243 for 24 hours. **(C)**, RT-qPCR analysis of TRIM56 mRNA level. **(D)**, Immunoblot analysis of α-tubulin, UCP1, and TRIM56 proteins, with their quantification. n=6 per group.
2. **G**, Male wild-type (WT) mice received intraperitoneally injections of either PBS or CL316243 (1 mg/kg/day) for 7 days. **(E)** Representative UCP1 and TRIM56 IHC images. Scale bar, 100μm. Quantitative analysis was shown on the right (n=6). **(F)** Representative immunoblot images of β-actin and TRIM56 proteins with quantification (n=6). **(G)** RT-qPCR analysis of TRIM56 level (n=6).

**H**, RT-qPCR analysis was performed to measure TRIM56 levels in the BAT and eWAT of mice subjected to 30°C housing and a three-day cold exposure at 4°C (n=6).

**I,** Representative immunoblot images of β-actin and TRIM56 proteins in the BAT and eWAT of mice mentioned in panel H, with its quantification was shown (n=6).

**J-K**, Mice were fed with a 3-month high fat diet or a 3-month standard control diet. **(J)**, RT-qPCR analysis was performed to measure TRIM56 levels in the BAT and eWAT (n=6). **(K)**, Representative immunoblot images of β-actin and TRIM56 proteins in the BAT and eWAT, with its quantification was shown (n=6).

Data are presented as mean ± SEM. *, *P˂*0.05, **, *P˂*0.01, ***, *P˂*0.001, and ****, *P˂*0.0001. ns, non-significant.

**
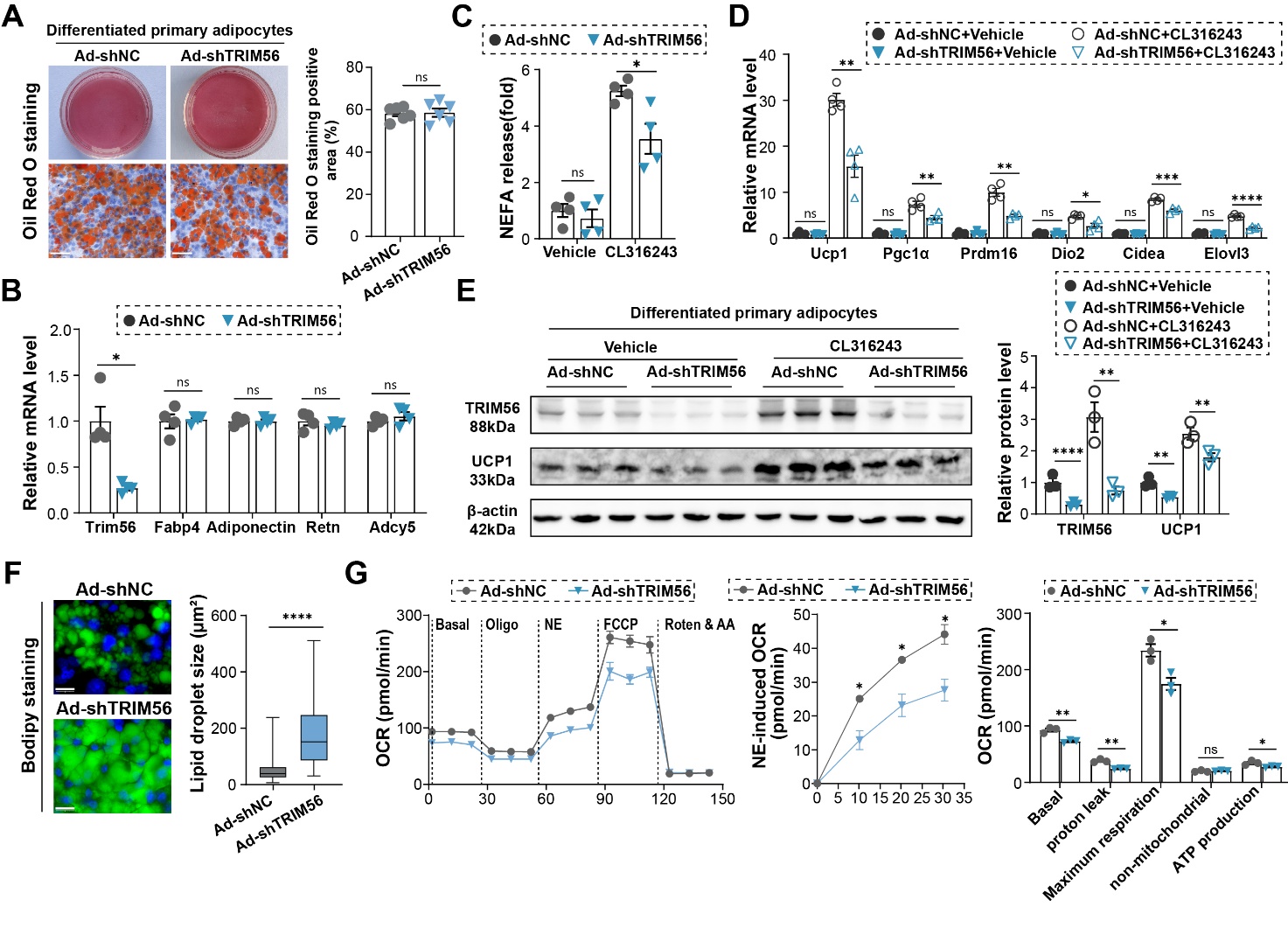
Extended Fig.2. Knockdown TRIM56 in adipocytes abolished CL316243-induced activation of beige adipocytes.**

SVF cells were isolated from iWAT and differentiated into beige adipocytes in the next 8d. Short hairpin RNA targeted TRIM56 or scramble control shRNA were delivered into mature adipocytes via adenovirus vectors. 24h later, cells were stimulated with 1um CL316243 for an additional 24 hours.

**A**, Represented Oil Red O staining images of mature beige adipocytes, which treated with TRIM56-knockdown adenovirus vectors or controls for 24h, with quantification of ORO staining positive area (right, n=6 per group).

**B**, RT-qPCR analysis of TRIM56 and specified adipocytes markers mRNA levels (n=4).

**C**, NEFA levels in the culture medium were measured. n=4 per group.

**D**, RT-qPCR analysis of indicated thermogenic genes (n=4).

**E**, Represented immunoblotting pictures of UCP1, TRIM56 and β-actin protein (left) with quantitative analysis (right, n=3).

**F**, Represented bodipy staining images of adenovirus treated adipocytes after 24 hours CL316243 stimulation with quantification of lipid droplet size (right, Ad-shNC: n=96; Ad-shTRIM56: n=47).

**G**, Mature adipocytes were treated with indicated stimulation. Various components of oxygen consumption rates (OCR) were measured (n=3).

Data are presented as mean ± SEM. *, *P˂*0.05, **, *P˂*0.01, ***, *P˂*0.001, and ****, *P˂*0.0001. ns, non-significant.

**
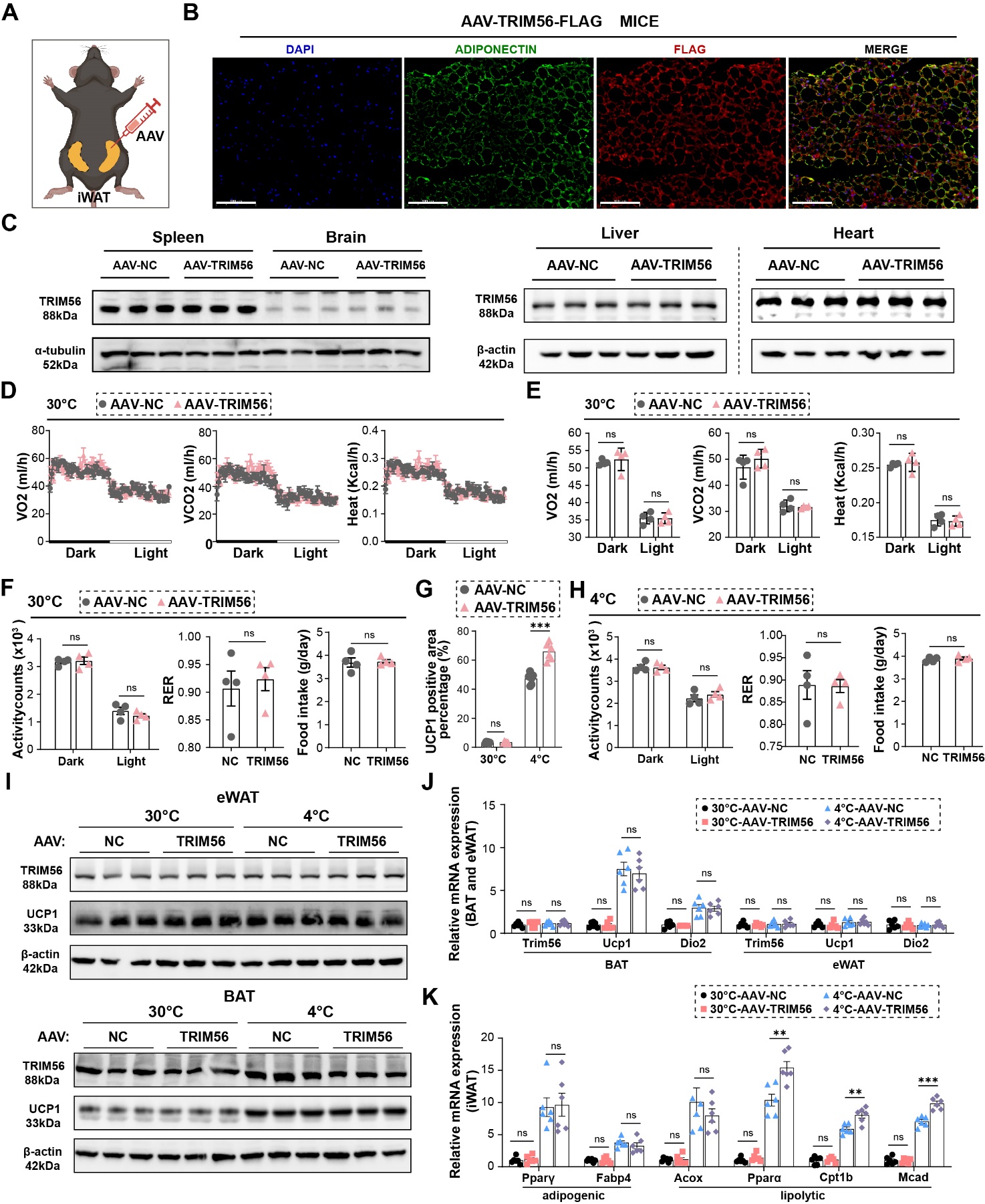
Extended Fig.3. TRIM56 overexpression in iWAT promotes cold-induced browning without affecting thermogenesis under thermoneutral conditions at 30**°C**.**

**A**, Schematic diagram of AAV subcutaneous Injection in Mice. This figure was created with BioRender.com/k23z013.

**B**, Representative immunofluorescence images showing FLAG (red), ADIPONECTIN (green), and DAPI (blue) staining in iWAT after 2 weeks of AAV-TRIM56-FLAG injection. Scale bar, 100μm.

**C**, Representative immunoblot images of TRIM56 expression in spleen, brain, liver, and heart tissues from mice injected with AAV-TRIM56-FLAG and controls.

1. **F**, Mice were kept at 30°C for 2 weeks. Metabolic rates were measured at the end of 30°C period. Analysis of O2 consumption, CO2 production, and energy expenditure **(D)** with corresponding quantification analysis **(E)**. Activity counts, food intake and respiratory exchange ratio (RER) of mice **(F)** were also analyzed. Dark/light bars represent 12-hour periods. n=4 per group.

**G**, Quantitative analysis was performed on the immunohistochemical results of UCP1 presented in Figure 3H.

**H-K**, Mice were housed at 30°C for 2 weeks, followed by 3 days of cold exposure. During the cold exposure phase, metabolic rates were measured. These results are shown in Figure 3. **(H)** Activity counts, food intake and respiratory exchange ratio (RER) of mice were also analyzed. **(I)** Representative immunoblot images of TRIM56, UCP1 and β-actin expression in BAT and eWAT. **(J)** RT-qPCR analysis of mRNA levels of *Ucp1, Dio2,* and *Trim56* in BAT and eWAT. **(K)** RT-qPCR analysis of mRNA levels of adipogenic and lipolytic genes in iWAT.

Data are presented as mean ± SEM. *, *P˂*0.05, **, *P˂*0.01, ***, *P˂*0.001, and ****, *P˂*0.0001. ns, non-significant.

**
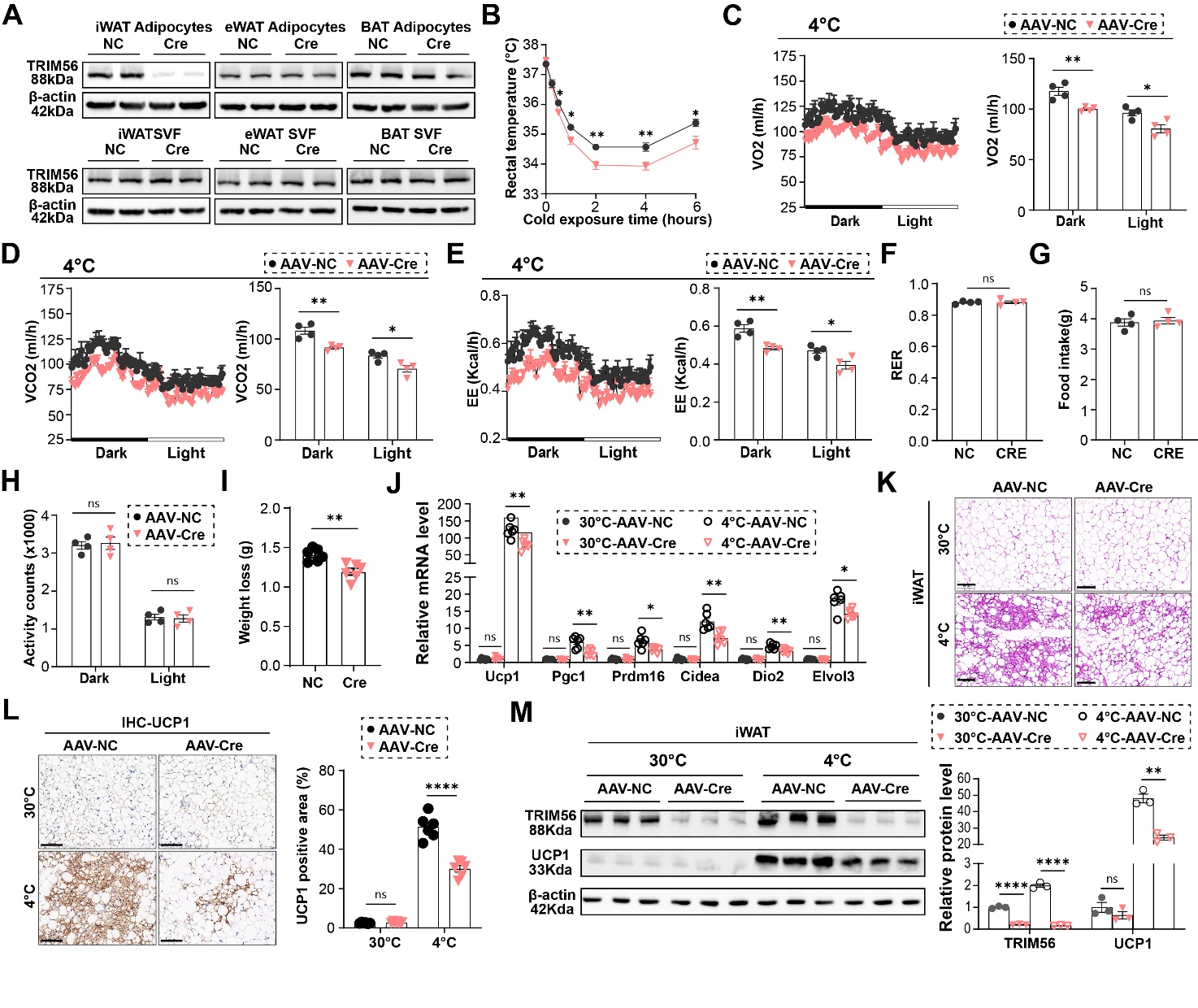
Extended Fig.4. TRIM56 deletion inhibits cold induced iWAT browning.**

Adiponectin-driven AAV-Cre or AAV-NC were locally injected subcutaneously into iWAT of TRIM56^fl/fl^ mice. The mice were then maintained in a thermoneutral environment for 3 weeks. After this period, they were divided into two groups: one group remained in the thermoneutral environment, while the other group was subjected to 3 days of cold exposure, with metabolic parameters assessed using the CLAMS system

**A**, Immunoblot analysis was performed to assess TRIM56 protein in fractionated adipocytes and stromal vascular fraction (SVF) cells from interscapular BAT, iWAT and eWAT of Cre-overexpressing mice and control littermates. β-actin protein was used as the loading control.

**B**, The mice underwent a cold challenge at 4°C, during which their rectal temperatures were monitored and recorded over the initial 6-hour period (n=6).

**C-E**, Analysis of O2 consumption **(C)**, CO2 production **(D)** and energy expenditure **(E)** over 24 hours (n=4).

**F-H**, Analysis of respiratory exchange ratio (RER) **(F),** food intake **(G)** and activity levels **(H)** during the CLAMS monitored period (n=4).

**I**, Weight loss in mice following 3 days of cold exposure (n=6).

**J**, RT-qPCR analysis of key thermogenic genes (n=6).

**K**, Representative hematoxylin and eosin (HE) stained sections of iWAT from the aforementioned mice. Scale bar, 100μm.

**L**, Representative immunostaining images of UCP1 in iWAT. The quantification was shown in the right (n=6). Scale bar, 100 μm.

**M**, Immunoblot analysis of UCP1, TRIM56, and β-actin proteins (left) with quantitative assessment (right, n=3).

Data are presented as mean ± SEM. *, *P˂*0.05, **, *P˂*0.01, ***, *P˂*0.001, and ****, *P˂*0.0001. ns, non-significant.

**
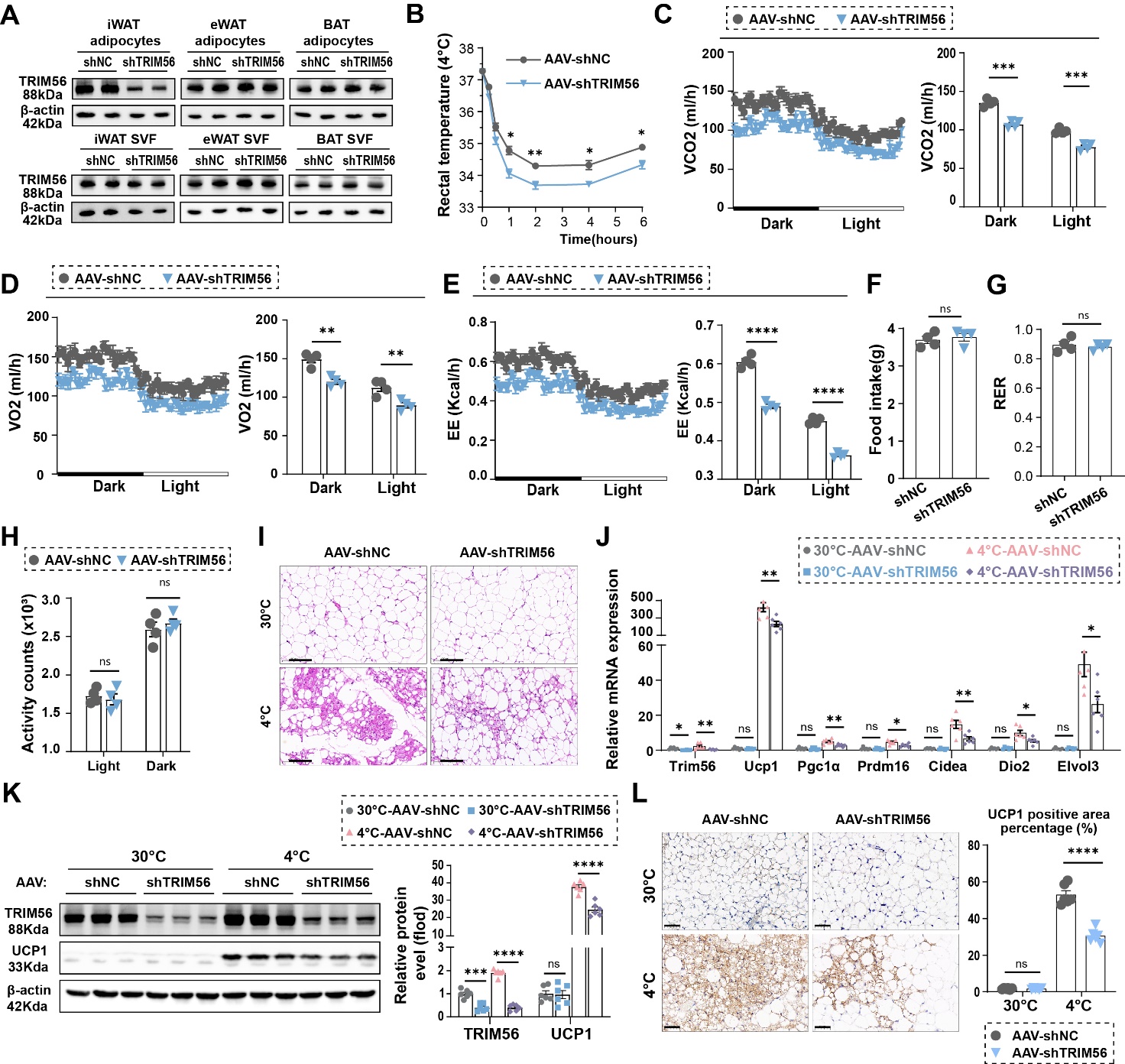
Extended Fig.5. TRIM56 deficiency inhibits cold induced iWAT browning.**

After acclimating at 30°C for 2 weeks, mice injected with AAV-shTRIM56 or AAV-shNC were transferred to 4°C for 3 days.

**A**, Representative immunoblot images showing TRIM56 expression in iWAT from mice injected with AAV-shTRIM56 and AAV-shNC vectors.

**B**, The mice underwent a cold challenge at 4°C, during which their rectal temperatures were monitored and recorded over the initial 6-hour period (n=8).

**C-E**, Analysis of O2 consumption, CO2 production and energy expenditure (heat). The dark/light bar means a 12 hours duration (n=4).

**F-H**, Quantification of food intake **(F)**, RER **(G)**, and activity levels **(H)** during the CLAMS monitored period (n=4).

**I**, Representative hematoxylin and eosin (HE) stained sections of iWAT from the aforementioned mice. Scale bar, 100μm.

**J**, RT-qPCR analysis of TRIM56, key thermogenic genes, adipogenic genes, and lipolytic genes (n=6).

**K**, Immunoblot analysis of UCP1, TRIM56, and β-actin proteins (left) with quantitative assessment (right, n=6).

**L**, Representative immunostaining images of UCP1 in iWAT. Quantitative analysis was shown on the right (n=6). Scale bar, 50 μm.

Data are presented as mean ± SEM. *, *P˂*0.05, **, *P˂*0.01, ***, *P˂*0.001, and ****, *P˂*0.0001. ns, non-significant.

**
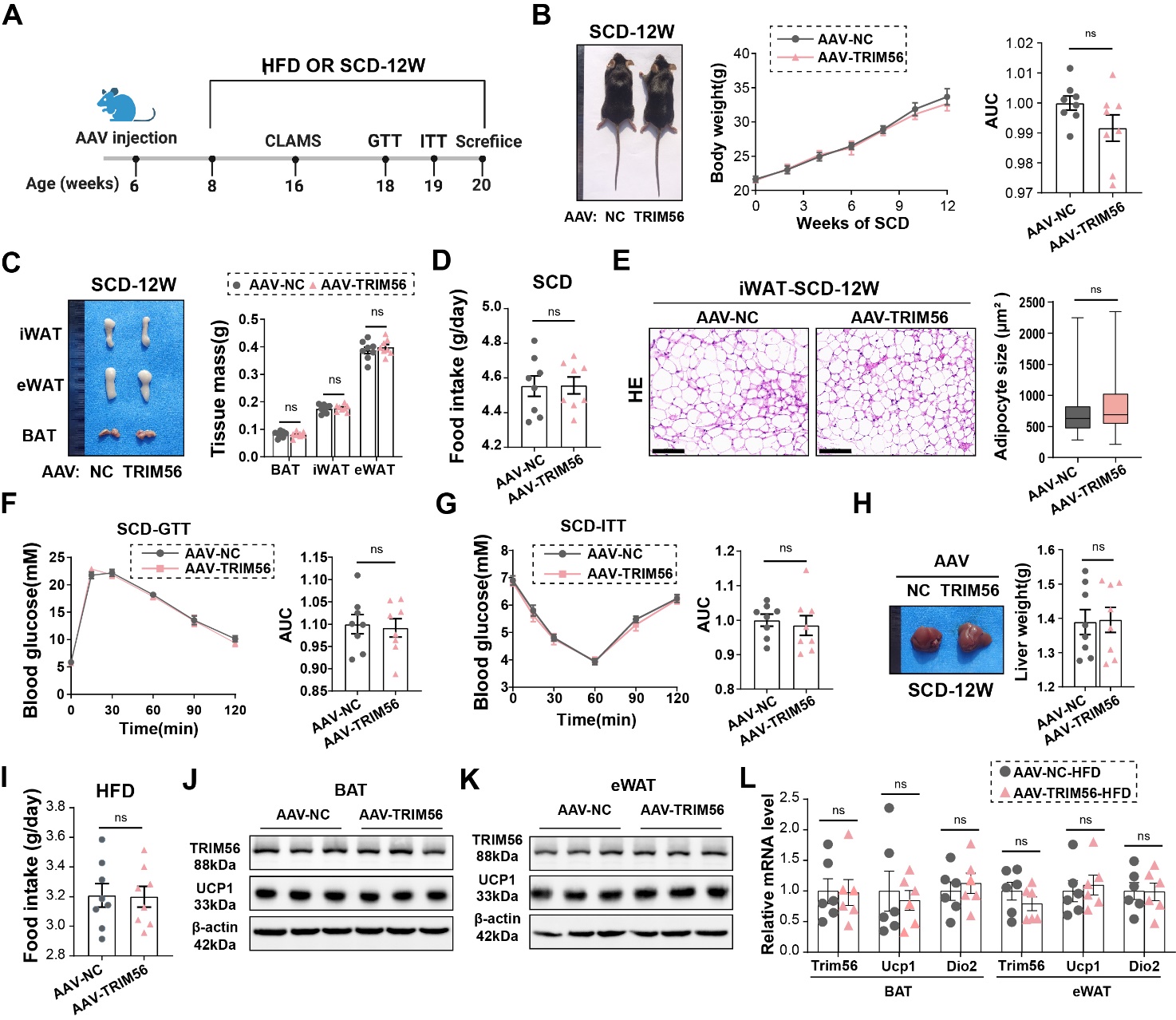
Extended Fig.6. TRIM56 overexpression in iWAT did not affect body weight under SCD. Figure 6A was created with BioRender.com/z61a006.**

**A**, The flowchart of experiment mentioned in Fig.4 and Extended Fig.6-7.

**B**, Represented gross morphology of mice fed with a 12-weeks SCD (left), the curve of body weight during the 12 weeks (middle) and its quantification (right, n=8).

**C**, Represented images of indicated tissues (iWAT, eWAT, BAT) from mice fed with 12-weeks SCD (left) and quantitative analysis of tissues wight (right, n=8).

**D**, Food-intake during the period fed with SCD (n=8).

**E**, Represented HE staining images of iWAT with the quantification of cell size (AAV-NC: n=178; AAV-TRIM56: n=136).

**F**, GTT of mice on SCD at specified time points (n=8).

**G**, ITT of SCD-fed mice at specified time points (n=8).

**H**, Represented liver images of mice fed with a 12-weeks SCD (left) and the quantitation of liver weights (right, n=8).

**I**, Food-intake during the period fed with HFD (n=8).

1. **L**, Mice were fed with a HFD for 12 weeks. These mice were mentioned in Figure 4. Representative immunoblot images of TRIM56, UCP1 and β-actin expression in BAT **(J)** and eWAT **(K)** . **(L)** RT-qPCR analysis of mRNA levels of *Ucp1, Dio2,* and *Trim56* in BAT and eWAT (n=6) .

Data are presented as mean ± SEM. *, *P˂*0.05, **, *P˂*0.01, ***, *P˂*0.001, and ****, *P˂*0.0001. ns, non-significant. SCD, standard control diet.

**
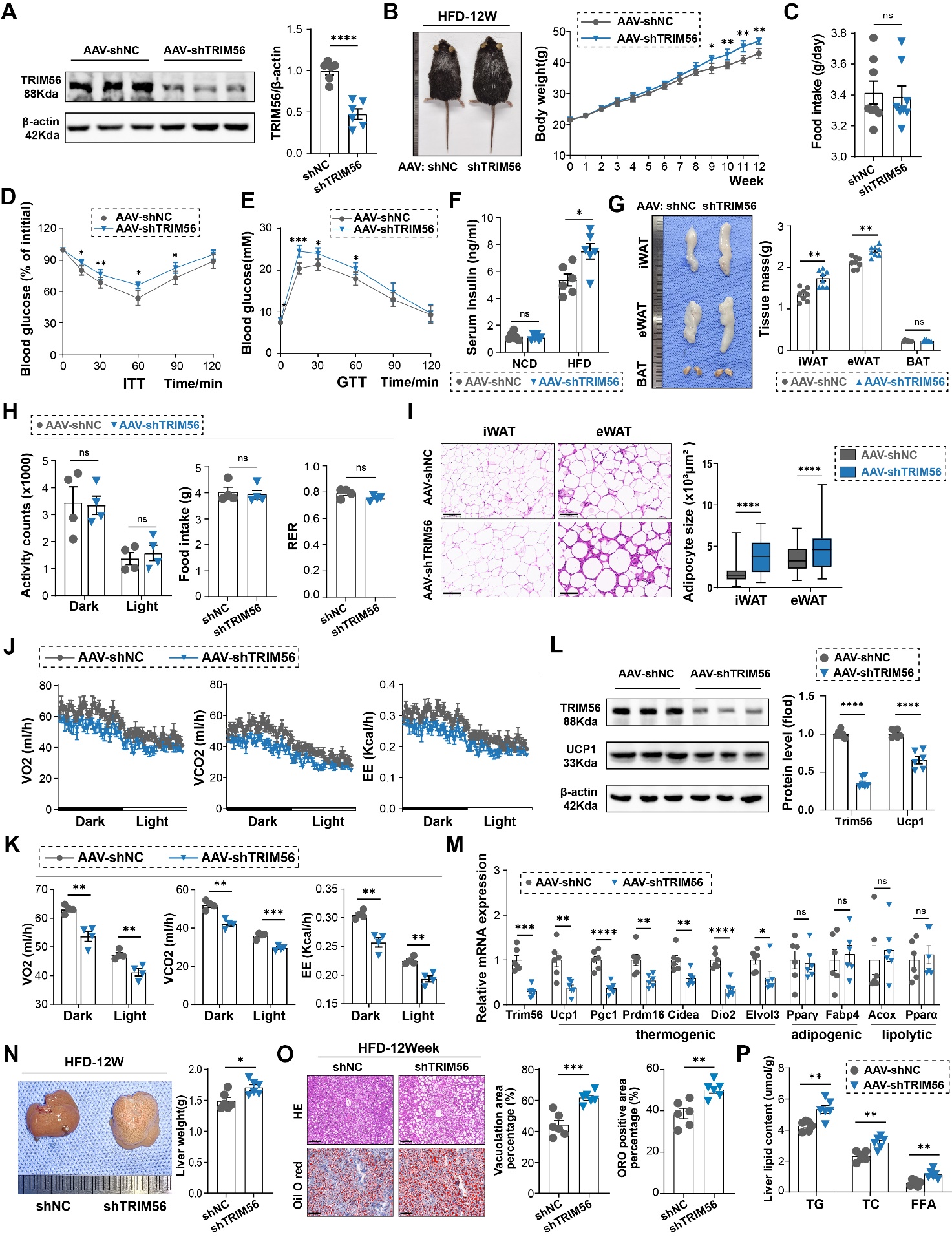
Extended Fig.7. TRIM56 loss-function leads to aggravation of HFD-induced obesity.**

Mice administered AAV-shNC and AAV-shTRIM56 were fed with HFD for 12 weeks.

**A**, Representative immunoblot image of TRIM56 and β-actin in iWAT serves to demonstrate the knockdown efficiency of specific AAV vectors (n=6) .

**B**, Represented gross morphology of mice fed with a 12-weeks HFD (left), the curve of body weight during the 12 weeks (right, n=8).

**C**, Quantification of food-intake (n=8).

**D-E**, ITT and GTT were conducted during the 10th and 11th weeks of HFD feeding (n=8).

**F**, Serum insulin levels after 12-week HFD feeding (n=6).

**G**, Represented images of indicated tissues (iWAT, eWAT) from mice fed with 12-weeks HFD (left) and quantitative analysis of tissues wight (right, n=8).

**H**, Quantification of activity levels, food intake and RER during the CLAMS monitored period (n=4).

**I**, Represented HE staining images of indicated adipose tissues (shNC-iWAT: 236 cells; shTRIM56-iWAT: 179 cells; shNC-eWAT: 153 cells; shTRIM56-eWAT: 117 cells).

**J**, The O2 consumption, CO2 production and energy expenditure of mice subjected to an 8-weeks HFD were analyzed (n=4). The dark/light bar equals 12 hours.

**K**, Quantitative analysis of parameters mentioned in panel J (n=4).

**L**, Immunoblotting analysis of TRIM56, UCP1 and β-actin protein (left), with its quantification (right, n=6).

**M**, RT-qPCR analysis of indicated thermogenic markers, adipogenic markers and lipolytic markers (n=6).

**N**, Represented liver images(left) and quantitative analysis of liver wights (right, n=6).

**O**, Represented HE and Oil Red O-stained liver section images. The quantitative results were shown in the right (n=6).

**P**, Liver lipid contents (TG, TC, FFA) were evaluated in mice after a 12-week high-fat diet regimen (n=6).

Data are presented as mean ± SEM. * *P˂*0.05, ** *P˂*0.01, *** *P˂*0.001 and **** *P˂*0.0001. ns, non-significant.

**
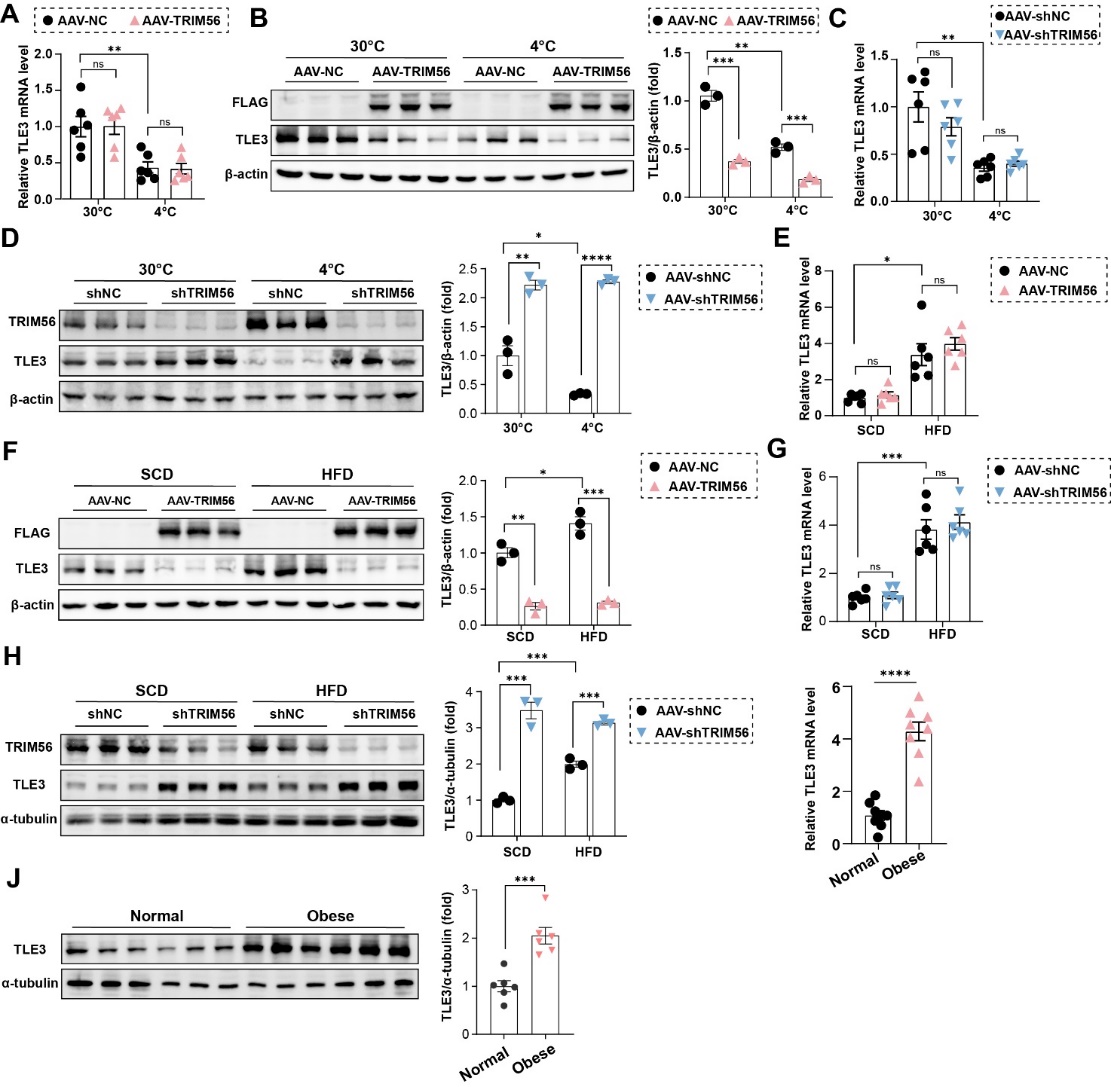
Extended Fig.8. Inverse Correlation Between TRIM56 and TLE3 Protein Levels.**

**A-H**, Mice injected with the specified AAV and housed under different conditions.

**A**, Mice were injected with AAV-TRIM56 or control virus. Then mice were housed under 30°C or 4°C. These mice were used in **Figure 3**. RT-qPCR analysis of TLE3 in iWAT (n=6).

**B**, The overexpressed TRIM56 was tagged with a FLAG label. Immunoblotting analysis of FLAG, TLE3 and β-actin proteins in the iWAT of mice, as shown in panels A (n=3).

**C-D**, These mice were used in **Extended Fig.5. (C)** RT-qPCR analysis of TLE3 in iWAT (n=6). **(D)** Immunoblotting analysis of TRIM56, TLE3, and β-actin proteins in the iWAT of mice (n=3).

**E-F**, These mice were used in **Fig.4. (E)** RT-qPCR analysis of TLE3 in iWAT of mice injected with the specified AAV and fed a HFD or SCD (n=6). **(F)** Immunoblotting analysis of FLAG, TLE3, and β-actin proteins in the iWAT of mice (n=3).

**G-H**, These mice were used in **Extended Fig.7.** **(G)** RT-qPCR analysis of TLE3 in iWAT of mice injected with the specified AAV and fed a HFD or SCD (n=6). **(H)** Immunoblotting analysis of TRIM56, TLE3, and α-tubulin proteins in the iWAT of mice (n=3).

**I**, RT-qPCR analysis of TLE3 in subcutaneous adipose tissue samples obtained from obese individuals with a BMI over 30 and those with a normal BMI (n=8).

**J**, Immunoblotting analysis of TLE3 and α-tubulin proteins in subcutaneous adipose tissue samples mentioned in panel I (n=6).

Data are presented as mean ± SEM. * *P˂*0.05, ** *P˂*0.01, *** *P˂*0.001 and **** *P˂*0.0001. ns, non-significant.

**Extended Table1. Primary antibodies used in this study.**

| Reagents | Source | Catalogue number |
| --- | --- | --- |
| β-actin | Abcam | ab8226 |
| UCP1 | Abcam | ab10983 |
| Flag | Sigma-Aldrich | F1804 |
| His | Proteintech | 66005-1-Ig |
| HA | Proteintech | 51064-2-AP |
| PGC1α | Abcam | ab191838 |
| α-tubulin | Abcam | ab7291 |
| TRIM56 | Abcam | ab154862 |
| Adiponectin | Proteintech | 21613-1-AP |
| HSL | Cell Signaling Technology | 4107 |
| Phospho-HSL (Ser660) | Cell Signaling Technology | 45804 |
| Phospho-HSL (Ser563) | Cell Signaling Technology | 4139 |
| ATGL | Cell Signaling Technology | 2138 |

| Reagents | Source |
| --- | --- |
| forward | 5′-GAGCTCTTACGCGTGCTAGCAGCTCTGCAAGCCTGACCTC-3′ |
| reverse | 5′-CAGTACCGGAATGCCAAGCTTGCTCTTCACGCCTGTCCGC-3′ |

**Extended Table2. Human UCP1 promoter primers**

**Extended Table 3. Oligonucleotide primers for qRT-PCR analysis.**

| Gene | Forward primer (5'-3') | Reverse primer (5'-3') |
| --- | --- | --- |
| Ucp1 | AGGCTTCCAGTACCATTAGGT | CTGAGTGAGGCAAAGCTGATTT |
| Dio2 | ATGGGACTCCTCAGCGTAG  AC | ACTCTCCGCGAGTGGACTT |
| Elovl3 | TTCTCACGCGGGTTAAAAAT  GG | GGCCAACAACGATGAGCAAC |
| Cidea | TCCTATGCTGCACAGATGAC  G | TGCTCTTCTGTATCGCCCAG  T |
| Ppargc1a | CCCTGCCATTGTTAAGACC | TGCTGCTGTTCCTGTTTTC |
| Prdm16 | ACACGCCAGTTCTCCAACCT  GT | TGCTTGTTGAGGGAGGAGGT  A |
| Adipoq | GAAGCCGCTTATGTGTATCG  C | GAATGGGTACATTGGGAACA  GT |
| Fabp4 | AAGGTGAAGAGCATCATAAC  CCT | TCACGCCTTTCATAACACATT  CC |
| Adcy5 | AACGCCAAGCAGGAGGATA  TG | CCCCGAGGATCTTAATCCGT  AA |
| Retn | AAGAACCTTTCATTTCCCCT  CCT | GTCCAGCAATTTAAGCCAAT  GTT |
| Ppara | AACATCGAGTGTCGAATATG  TGG | CCGAATAGTTCGCCGAAAGA  A |
| 18S | TTGACGGAAGGGCACCACCAG | GCACCACCACCCACGGAATCG |
| Acox | AAATATGCCCAGGTGAAGCC | CACTGTATCGAATGGCAATGG |
| Pparγ | TCGCTGATGCACTGCCTATG | GAGAGGTCCACAGAGCTGATT |
| TLE3 | ATGCAGCGCCATTATGTGATG | CAGTCTCTTCGCAATCTCTGTC |
| Trim56 | AAGACTCCTCCCCAACTCTG | GGCAATAGGTATGTAGGCATGG |
| TLE3(human) | TATCCGCAGGGCAGACATC | GTTTGCCAGCTTGTCGTACTC |
| Trim56(human) | GCCTGCATACCTACTGCCAAG | GCAGCCCATTGACGAAGAAGT |
| β-actin(human) | GGCACCCAGCACAATGAA | GGAAGGTGGACAGCGAGG |
| Mcad | AACACAACACTCGAAAGCGG | TTCTGCTGTTCCGTCAACTCA |
| Cpt1b | GACTTCCGGCTTAGTCGGG | GAATAAGGCGTTTCTTCCAGGA |

**Extended Table4. The protein sequences of TRIM56 and TLE3 used for structural prediction.**

| TRIM56 | MVSHGSSPSLLEALSSDFLACKICLEQLRAPKTLPCLHTYCQDCLAQLADGGRVRCPECRETVPVPPEGVASFKTNFFVNGLLDLVKARACGDLRAGKPACALCPLVGGTSTGGPATARCLDCADDLCQACADGHRCTRQTHTHRVVDLVGYRAGWYDEEARERQAAQCPQHPGEALRFLCQPCSQLLCRECRLDPHLDHPCLPLAEAVRARRPGLEGLLAGVDNNLVELEAARRVEKEALARLREQAARVGTQVEEAAEGVLRALLAQKQEVLGQLRAHVEAAEEAARERLAELEGREQVARAAAAFARRVLSLGREAEILSLEGAIAQRLRQLQGCPWAPGPAPCLLPQLELHPGLLDKNCHLLRLSFEEQQPQKDGGKDGAGTQGGEESQSRREDEPKTERQGGVQPQAGDGAQTPKEEKAQTTREEGAQTLEEDRAQTPHEDGGPQPHRGGRPNKKKKFKGRLKSISREPSPALGPNLDGSGLLPRPIFYCSFPTRMPGDKRSPRITGLCPFGPREILVADEQNRALKRFSLNGDYKGTVPVPEGCSPCSVAALQSAVAFSASARLYLINPNGEVQWRRALSLSQASHAVAALPSGDRVAVSVAGHVEVYNMEGSLATRFIPGGKASRGLRALVFLTTSPQGHFVGSDWQQNSVVICDGLGQVVGEYKGPGLHGCQPGSVSVDKKGYIFLTLREVNKVVILDPKGSLLGDFLTAYHGLEKPRVTTMVDGRYLVVSLSNGTIHIFRVRSPDS |
| --- | --- |
| **TLE3** | MYPQGRHPAPHQPGQPGFKFTVAESCDRIKDEFQFLQAQYHSLKVEYDKLANEKTEMQRHYVMYYEMSYGLNIEMHKQTEIAKRLNTILAQIMPFLSQEHQQQVAQAVERAKQVTMTELNAIIGQQQLQAQHLSHATHGPPVQLPPHPSGLQPPGIPPVTGSSSGLLALGALGSQAHLTVKDEKNHHELDHRERESSANNSVSPSESLRASEKHRGSADYSMEAKKRKAEEKDSLSRYDSDGDKSDDLVVDVSNEDPATPRVSPAHSPPENGLDKARSLKKDAPTSPASVASSSSTPSSKTKDLGHNDKSSTPGLKSNTPTPRNDAPTPGTSTTPGLRSMPGKPPGMDPIGIMASALRTPISITSSYAAPFAMMSHHEMNGSLTSPGAYAGLHNIPPQMSAAAAAAAAAYGRSPMVSFGAVGFDPHPPMRATGLPSSLASIPGGKPAYSFHVSADGQMQPVPFPHDALAGPGIPRHARQINTLSHGEVVCAVTISNPTRHVYTGGKGCVKIWDISQPGSKSPISQLDCLNRDNYIRSCKLLPDGRTLIVGGEASTLTIWDLASPTPRIKAELTSSAPACYALAISPDAKVCFSCCSDGNIAVWDLHNQTLVRQFQGHTDGASCIDISHDGTKLWTGGLDNTVRSWDLREGRQLQQHDFTSQIFSLGYCPTGEWLAVGMESSNVEVLHHTKPDKYQLHLHESCVLSLKFAYCGKWFVSTGKDNLLNAWRTPYGASIFQSKESSSVLSCDISADDKYIVTGSGDKKATVYEVIY |
